# A prototypical non-malignant epithelial model to study genome dynamics and concurrently monitor micro-RNAs and proteins *in situ* during oncogene-induced senescence

**DOI:** 10.1186/s12864-017-4375-1

**Published:** 2018-01-10

**Authors:** Eirini-Stavroula Komseli, Ioannis S. Pateras, Thorbjørn Krejsgaard, Konrad Stawiski, Sophia V. Rizou, Alexander Polyzos, Fani-Marlen Roumelioti, Maria Chiourea, Ioanna Mourkioti, Eleni Paparouna, Christos P. Zampetidis, Sentiljana Gumeni, Ioannis P. Trougakos, Dafni-Eleftheria Pefani, Eric O’Neill, Sarantis Gagos, Aristides G. Eliopoulos, Wojciech Fendler, Dipanjan Chowdhury, Jiri Bartek, Vassilis G. Gorgoulis

**Affiliations:** 1 Molecular Carcinogenesis Group, Department of Histology and Embryology, School of Medicine, National & Kapodistrian University of Athens, 75 Mikras Asias St, GR-11527 Athens, Greece; 2 Department of Immunology and Microbiology, University of Copenhagen, Blegdamsvej 3c, DK-2200 Copenhagen, Denmark; 3 Department of Biostatistics and Translational Medicine, Medical University of Lodz, 15 Mazowiecka St. 92-215, Lodz, Poland; 4 Biomedical Research Foundation of the Academy of Athens, 4 Soranou Ephessiou St, GR-11527 Athens, Greece; 5 Department of Cell Biology and Biophysics, Faculty of Biology, National & Kapodistrian University of Athens, GR-15784 Athens, Greece; 6 CRUK/MRC Institute for Radiation Oncology, Department of Oncology, University of Oxford, Oxford, OX3 7DQ UK; 7 Department of Biology, School of Medicine, National & Kapodistrian University of Athens, 75 Mikras Asias St, GR-11527 Athens, Greece; 8 Institute of Molecular Biology and Biotechnology, Foundation for Research & Technology-Hellas, GR-70013 Heraklion, Crete Greece; 9 Department of Radiation Oncology, Dana-Farber Cancer Institute, 450 Brookline Ave, Boston, MA 02215 USA; 10 Harvard Medical School, 25 Shattuck St, Boston, MA 02115 USA; 11 Genome Integrity Unit, Danish Cancer Society Research Centre, Strandboulevarden 49, DK-2100 Copenhagen, Denmark; 12 Institute of Molecular and Translational Medicine, Faculty of Medicine and Dentistry, Palacky University, Hněvotínská, 1333/5, 779 00 Olomouc, Czech Republic; 13 Department of Medical Biochemistry and Biophysics, Karolinska Institute, Science for Life Laboratory, Division of Translational Medicine and Chemical Biology, SE-171 77 Stockholm, Sweden; 14 Faculty of Biology, Medicine and Health, University of Manchester, Manchester Academic Health Science Centre, Wilmslow Road, Manchester, M20 4QL UK

**Keywords:** *In situ* hybridization, Micro-RNAs, Replication stress, Oncogene-induced senescence, CDC6, SenTraGorTM, DNA damage response, R loops, rDNA, Cancer

## Abstract

**Background:**

Senescence is a fundamental biological process implicated in various pathologies, including cancer. Regarding carcinogenesis, senescence signifies, at least in its initial phases, an anti-tumor response that needs to be circumvented for cancer to progress. Micro-RNAs, a subclass of regulatory, non-coding RNAs, participate in senescence regulation. At the subcellular level micro-RNAs, similar to proteins, have been shown to traffic between organelles influencing cellular behavior. The differential function of micro-RNAs relative to their subcellular localization and their role in senescence biology raises concurrent *in situ* analysis of coding and non-coding gene products in senescent cells as a necessity. However, technical challenges have rendered *in situ* co-detection unfeasible until now.

**Methods:**

In the present report we describe a methodology that bypasses these technical limitations achieving for the first time simultaneous detection of both a micro-RNA and a protein in the biological context of cellular senescence, utilizing the new commercially available SenTraGor^TM^ compound. The method was applied in a prototypical human non-malignant epithelial model of oncogene-induced senescence that we generated for the purposes of the study. For the characterization of this novel system, we applied a wide range of cellular and molecular techniques, as well as high-throughput analysis of the transcriptome and micro-RNAs.

**Results:**

This experimental setting has three advantages that are presented and discussed: i) it covers a “gap” in the molecular carcinogenesis field, as almost all corresponding *in vitro* models are fibroblast-based, even though the majority of neoplasms have epithelial origin, ii) it recapitulates the precancerous and cancerous phases of epithelial tumorigenesis within a short time frame under the light of natural selection and iii) it uses as an oncogenic signal, the replication licensing factor CDC6, implicated in both DNA replication and transcription when over-expressed, a characteristic that can be exploited to monitor RNA dynamics.

**Conclusions:**

Consequently, we demonstrate that our model is optimal for studying the molecular basis of epithelial carcinogenesis shedding light on the tumor-initiating events. The latter may reveal novel molecular targets with clinical benefit. Besides, since this method can be incorporated in a wide range of low, medium or high-throughput image-based approaches, we expect it to be broadly applicable.

**Electronic supplementary material:**

The online version of this article (10.1186/s12864-017-4375-1) contains supplementary material, which is available to authorized users.

## Background

For almost half a century RNA was considered just the coupler between DNA and protein production. On the other hand, proteins represented the main workhorse of the cell. This view has dramatically changed over the last years, as regulatory RNAs emerged as a versatile component of the molecular machinery interacting with both DNA and proteins “shaping” gene expression and protein function, respectively [1, 2]. Mounting evidence shows that this bidirectional interplay plays a vital role in a variety of cellular responses, such as senescence; the latter involved in the pathogenesis of various diseases, including cancer.

Tumors are mixed tissues composed of cancer cells, with a tremendous phenotypic plasticity (tumor heterogeneity), and normal recruited cells that form the tumor-associated stroma [3, 4]. Under the microscope, a spatial organization of malignant and surrounding stromal cells is evident, implying a functional interplay between the various cell populations. Indeed, tumor development is mediated through a continuous “cross talk” between intra-cellular molecular pathways and inter-cellularly among cancer cells and the surrounding adjacent stroma [5]. The introduction of “-omics” increased our perception of the underlying processes occurring during carcinogenesis at a scale that has never been feasible before. However, the results of high-throughput analyses are in fact heterogeneous signals deriving from all the different cellular elements that comprise tumors. Thus, the molecular signature of distinct, relatively small tumor cell populations can be dramatically reduced or even lost, eliminating the ability to comprehend their contribution to cancer development. Hence, *in situ* detection of biomolecules rises as a crucial necessity to interrogate the mechanistic aspects of carcinogenesis.

*In situ* hybridization (ISH) and immune-localization assays are valuable tools for detecting spatial features of the transcriptional and translational machineries. ISH is among the most frequent techniques employed for the study of gene coding and non-coding RNAs (ncRNAs), including micro-RNAs (miRs). miRs have been demonstrated to play pivotal roles in multiple cell-fate decisions [6–12]. They are short, ~22 nucleotides in length, highly conserved and, although abundant, escaped notice until 1993, when they were first reported by Lee, Freinbaum and Ambros [13]. Biogenesis of miRs begins with the generation of long primary transcripts, called primary miRs (pri-miRs), by RNA polymerase II (RNA pol II) **(**Additional file 1: Figure S1). Subsequently, pri-miRs are processed by Drosha, a class 2 RNase III, forming the precursor miRs (pre-miRs) that are exported in the cytoplasm, by exportin-5. In the cytoplasm Dicer, a class 3 RNase III, produces the mature single strand that is loaded on the RISC (RNA-induced silencing complex) effector complex [14, 15]. Pairing between miR and its target mRNA facilitated by Argonaute, a core component of RISC, promotes post-transcriptional down-regulation by cleavage and degradation **(**Additional file 1: Figure S1). Besides their cytoplasmic localization and role, a number of miRs have been discovered to function in the nucleus [16]. The picture becomes even more fascinating, as miRs have been detected in the mitochondria affecting their dynamics, further underscoring the importance of monitoring miRs spatial distribution [17, 18] **(**Additional file 1: Figure S1 and Additional file 2: Table S1).

Of particular interest is the role of miRs in cellular senescence [19–22] (Additional file 3: Table S2). The latter is a fundamental biological process involved in tissue homeostasis during normal development, and implicated in a broad spectrum of pathologies, including cancer [23–26]. In mammalian cells two types of cellular senescence have been recognized; namely, replicative senescence (RS) that occurs after certain number of cell divisions due to telomere attrition and stress-induced premature senescence (SIPS) [27]. SIPS is telomere-independent and represents an acute response to a wide range of stressful stimuli long before telomere erosion appears [27]. A decade ago, we and others demonstrated that oncogenes trigger SIPS and that this response constitutes a potent anti-tumor barrier [28–34]. This type of senescence is referred to as “oncogene-induced senescence” (OIS) [35] and according to the oncogene-induced DNA damage model for cancer development, OIS must be bypassed for tumor progression [36].

Even though senescence affects a variety of cell types, the in vitro cellular models recurrently used to study the phenomenon of OIS are almost always fibroblast-based (Additional file 4: Table S3). As most common malignancies are of epithelial origin, the lack of non-malignant epithelial-derived models constitutes a major “gap” in the field. The basic grounds for the rareness of non-malignant epithelial-based cellular systems are: **i)** the difficulty in maintaining the specialized epithelial features during cultivation, rendering de-differentiation a frequent outcome [37], and **ii)** the unsuccessful immortalization with ectopic expression of human telomerase reverse transcriptase (hTERT) in epithelial cells in contrast to fibroblasts [38, 39].

Additionally, given the significance of miRs in senescence biology and their diverse intracellular localization **(**Additional file 1: Figure S1 and Additional file 2: Table S1) we were surprised to notice that there are no *in situ* studies of miRs in senescent cells **(**Additional file 3: Table S2). All the reports suffer from the limitation of analyzing miRs by qRT-PCR (quantitative reverse-transcription polymerase chain reaction) or micro-arrays of RNA isolated from cell cultures or tissues, independently scanned for senescence markers; thus lacking cellular specificity in cases of heterogeneity **(**Additional file 3: Table S2). The main reason behind the absence of *in situ* miR studies relies on restraints of the currently applied method for detecting senescence. The assay universally used, senescence-associated β-galactosidase (SA-β-Gal) assay, is based on the activity of β-galactosidase at suboptimal pH (pH 6.0). Among the steps incorporated in this method are: **i)** instant application on fresh cells/tissues to avoid loss of enzymatic activity; thus rendering prior RNA detection unfeasible, and **ii)** a lengthy incubation of 12–16 h at 37 °C in a complex ion buffer of pH 6.0 containing magnesium ions (Mg^+2^) [40], conditions which negatively affect RNA stability [41–45]. Although miRs are more stable than the larger RNA species [46–48], they eventually undergo degradation, as well [49, 50]. As recently shown, in urine samples stored for prolonged time at room temperature their quantity decreases on average to 81% of their initial levels at 24-h intervals [51]. Another potential source of RNA degradation, during application of the aforementioned method, may be the presence of RNases that are known to be very stable enzymes and cannot be inactivated by the fixative procedure applied by Dimri et al. [40, 52]. As a result, prolonged incubations at 37 °C may allow the decay mechanisms to act in parallel exerting their activity.

We recently reported a universally applicable hybrid histo−/immunohisto-chemical method that can bypass all the aforementioned constraints of detection of senescent cells [53]. This is a non-enzymatic assay based on the property of a novel reagent, termed GL13, to detect lipofuscin, a non-degradable metabolic by-product that is considered a “hallmark” of senescence [54]. In the present study, we have developed a method that enables the simultaneous detection of gene coding (proteins) and non-coding (miRs) products in GL13-reactive senescent cells *in situ*. We have applied this methodology in an epithelial cell model that over-expresses the replication licensing factor CDC6 (Cell division cycle 6) in an inducible manner. We show that this cell system recapitulates the whole spectrum of epithelial carcinogenesis from the non-malignant stage to oncogene-mediated activation of the anti-tumor barrier of senescence, followed by escape and the production of aggressive clones with features of epithelial to mesenchymal transition (EMT).

## Results

### The underlying principle for generating a CDC6-based non-malignant human epithelial model to study oncogene-induced senescence

According to a carcinogenesis model we have proposed, activated oncogenes disrupt normal DNA replication provoking replication stress that in turn triggers the DNA damage response (DDR) pathway, promoting genomic instability and cancer development [36]. An early feature of this process is that specific loci of the genome, called common fragile sites (CFS) are targeted resulting in breaks, gaps and rearrangements [55, 56]. The carcinogenic process we describe is not an undemanding procedure as incipient cancer cells need to evade the anti-tumor barriers of apoptosis and senescence to evolve into full-blown malignant cells [26, 36]. Thus, roughly the model can be divided into two phases: the first is typified by the activation of anti-tumor barriers, and the second is characterized by escape from the tumor-suppressor “blockade” driving cancer progression. The type of anti-tumor response elicited, apoptosis or senescence, is determined largely by the cellular context, however almost all studies, as already mentioned, examine the response to oncogene activation in mesenchymal-based cellular settings (Additional file 4: Table S3). Regarding the “escape from the tumor-suppressor barriers”, the cancer biology field relies mainly on genetic manipulation of potential anti-tumor network players and monitoring of barrier circumvention. Although this experimental approach is useful in drawing conclusions about the role of a particular factor, it lacks the element of “natural evolution”.

**Fig. 1:**
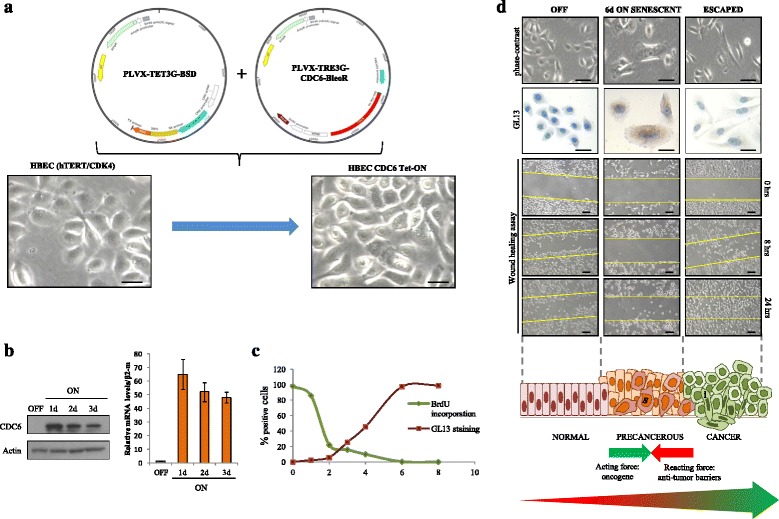
Epithelial cancer evolution experiment (ECEE) in a CDC6-expressing non malignant epithelial model. **a**-**b** Generation and validation of the HBEC CDC6 Tet-ON cellular system. **a** Schematic representation of the lentiviral vectors (PLVX-Tet3G-BSD and PLVX-TRE3G-CDC6-BleoR) utilized for CDC6 transduction in HBECs (see details in “Methods” section). HBECs preserve their epithelial phenotype upon ectopic expression of hTERT and mutant CDK4 for immortalization. Inverted-phase contrast microscopy showed preservation of the epithelial morphology in HBECs upon transduction with lentiviruses, followed by treatment with antibiotics in order to isolate clones with inducible CDC6 over-expression. Scale bar: 10 μm. **b** Efficiency of CDC6 induction was confirmed both at protein (western blot) and mRNA (qRT-PCR) levels at the indicated time points. **c** Plot showing inverse relationship between proliferation rate, as measured by BrdU incorporation overnight, and senescent phenotype, as assessed by GL13 staining [53], during the time course of CDC6 induction. **d** Morphological and kinetic features observed by inverted-phase contrast microscopy (Scale bar: 20 μm), GL13 staining (Scale bar: 20 μm) and wound healing assay (Scale bar: 80 μm) at specific time points of ECEE representing normal, precancerous and cancerous stages of tumorigenesis. Non-induced cells (“OFF”) are near normal, 6-day induced cells recapitulate precancerous lesions, where senescent cells are evident (dark orange cells in cartoon [s]), and cells that have escaped from senescence (“ESCAPED”) share the invasive characteristics of cancer cells (dark green cells in cartoon [i]). A continuous counter-interaction between the oncogenic acting force and the anti-tumor reacting force (senescence) takes places at the precancerous stage, leading eventually to the prevalence of the tumor promoting effect, bypass of senescence and emergence of cancer cells [36]

The requirement of a non-malignant model to perform an *epithelial cancer evolution experiment* (chronic expression of the oncogene without genetic intervention upon its activation) led us to utilize human bronchial epithelial cells (HBECs) as a platform to generate an oncogene doxycycline-inducible (Tet-ON) cellular system. Immortalization with combined expression of hTERT and ectopic mutant cyclin-dependent kinase 4 (CDK4) was successful in order to bypass p16^INK4A^-induced premature growth arrest **(**Additional file 5: Figure S2), maintaining the epithelial phenotype of the cells [57–59]. Further genetic manipulations to develop the inducible system also did not affect the epithelial characteristics of this model (Fig. 1a). We employed the replication licensing factor CDC6 as an inducible oncogene for the following reasons: **i)** it is a key component of the replication licensing machinery, found to be frequently deregulated in cancer from its earliest stages [60–62], and **ii)** when over-produced it displays a multi-functional facet by compromising the replication process (re-replication: a form of replication stress) triggering genomic instability [33, 60, 63, 64], and acquiring properties of a transcriptional regulator affecting: **a)** negatively the expression of the nodal tumor-suppressors loci, *INK4/ARF* (encoding p16^INK4A^, ARF and p15^INK4B^) and *CDH1* (encoding E-cadherin) [62, 63, 65] (Additional file 6: Figure S3), and **b)** positively that of *rDNA* (ribosomal DNA)*,* most probably impinging on RNA dynamics [66].

### Epithelial cancer evolution experiment (ECEE)

**Fig. 2:**
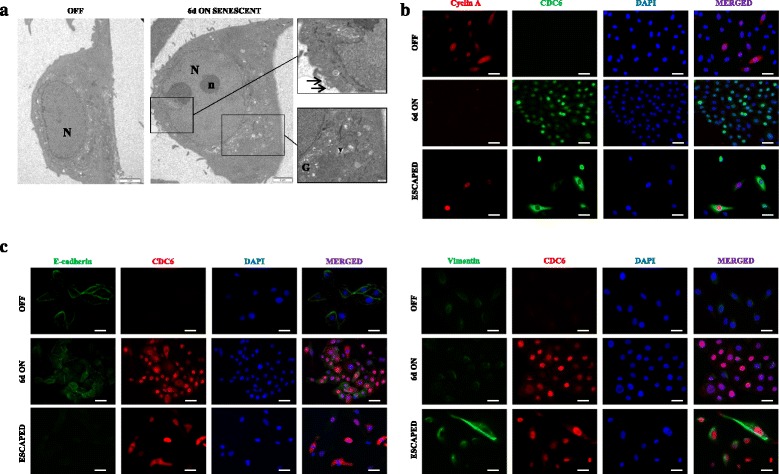
Phenotypic characterization of HBEC CDC6 Tet-ON system during ECEE. **a** Ultrastructural analysis of 6 days induced cells revealed flattened cellular morphology, with enlarged nucleus (N), enhanced protein-synthesis, as visualized by double nucleoli (n) and extensive Golgi apparatus (G), and formation of extracellular vesicles (v). Arrows depict extracellular vesicles. Scale bars 2 μm and 500 nm. **b** IF analysis of the proliferative marker (Cyclin A) and CDC6 showed double positive cells only after escape from senescence. Scale bar: 15 μm. **c** IF for an epithelial marker (E-cadherin) or a mesenchymal marker (Vimentin) along with CDC6 indicated EMT in the “escaped” cells. IF for CDC6 showed ubiquitous and uniform expression in the induced cells (“ON” and “ESCAPED”). Scale bar: 20 μm

Subsequent to validating the efficacy of CDC6 induction in the newly formed HBEC CDC6 Tet-ON system (Fig. 1b), we constitutively expressed it at levels relevant to those of tumor samples and monitored the cellular behavior over time. Stimulation of CDC6 resulted in a progressive decrease of cell proliferation, as depicted by reduced BrdU (bromodeoxyuridine) incorporation (Fig. 1c), that ceased after 6 days of induction. As proliferation diminished, the cells after a 3-day induction gradually acquired a senescent phenotype, as depicted by GL13 staining [53], that peaked at day 6 (Fig. 1c-d and Additional file 7: Figure S4). This finding is in line with a previous study where we showed that forced expression of CDC6 in human fibroblasts, induced senescence in a DDR-dependent manner [33]. Compared to fibroblasts, we were surprised by the robustness of this reaction in HBECs. This is quite intriguing given that senescence seems to be the predominant stress response program in fibroblasts, whereas apoptosis is believed to be the prevalent route in epithelial cells [67]. However, a recent report showing CDC6-mediated inhibition of apoptosome formation through its binding onto cytochrome *c*-activated Apaf-1 [68], probably explains why senescence emerges as the only tumor suppressor mechanism in our system. From a morphological point of view, microscopical analysis revealed enlarged and flattened cellular shapes, occasionaly multinucleated (Fig. 1d and Additional file 7: Figure S4), without the appearance of senescence associated heterochromatin foci (SAHF). Notably, this feature is observed during irreversible senescence in cells with an intact p16^INK4A^/Rb pathway, justifying its absence in our system [69]. On the other hand, the senescent cells presented extensive vacuolation, as well as the formation of extracellular vesicles indicative of increased secretoty activity, which may be related to the senescence associated secretory phenotype (SASP), the so called “dark side” of the senescent program accountable for its protumorigenic properties [26] (Fig. 2a). After a protracted stalled growth phase (around a month), when all cells were senescent and uniformaly expressed CDC6 (Figs. 1d and 2b-c and Additional file 7: Figure S4), a fraction of proliferating cells emerged with distinct morphological features compared to those of the “OFF” state. These cells, from now on termed “escaped”, showed traces of lipofuscin during the first cell divisions, proving that they came from senescent cells, while they were negative for GL13 after several passages and serial dilutions of the non-degradable metabolic by-product (Fig. 1d and Additional file 7: Figure S4). Moreover, they were double positive for CDC6 and Cyclin A, a well characterized cell proliferation marker (Fig. 2b). Unexpectedly, they attained a spindle morphology resembling that of mesenchymal cells (Fig. 1d and Additional file 7: Figure S4), insinuating an EMT, an embryonic program implicated in cancer invasion and progression [70, 71]. In accordance, E-cadherin, a fundamental adhesion molecule of epithelial tissues, was lost in the “escaped” cells, identifying a cardinal feature of the EMT program, whereas vimentin, a mesenchymal marker, increased (Fig. 2c) [71]. From a kinetic angle, carrying out a migration assay (wound healing assay) we observed that the “escaped” cells migrated significantly faster than the non-induced ones, whereas the senescent cells were completely still (Fig. 1d). To sum up, we developed an in vitro oncogene-regulated non-malignant human epithelial model that could compellingly recapitulate the precancerous and cancerous phases of the model we proposed (Fig. 1d) [36].

### Mechanistic insights into the senescent and escape phases of the ECEE

To gain a mechanistic view underlying the phenotypic phases we described (Figs. 1-2 and Additional file 7: Figure S4) we followed a dual approach: **i)** based on our prior knowledge, we hypothesized and examined, whether senescence was DRR-induced [33, 36, 60, 72–74] and **ii)** we performed high-throughput analyses of transcriptome and miRs. We performed transcriptome analysis at two time points, one at day 3 (initiation of the senescent phase) and a second during the “escaped” phase (evolution of the protumorigenic phase) to unravel potential cancer initiating and driver events, respectively, while we added another time point at day 6 (full senescent phenotype) in order to reveal a potential miR signature of the senescent cells.

**Fig. 3:**
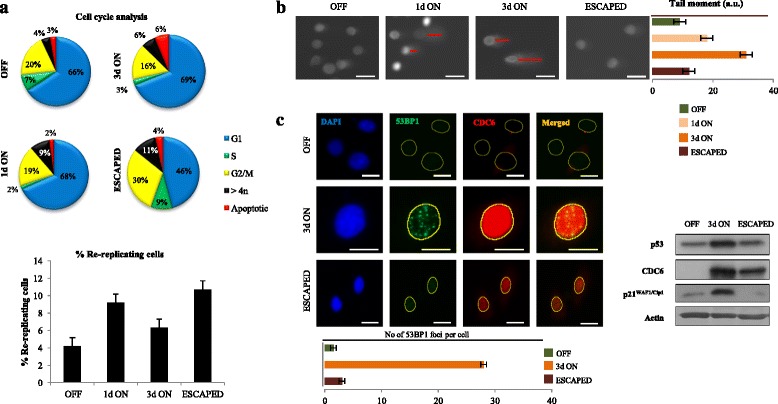
DNA damage and DDR activation upon CDC6 induction are reduced in the “escaped” cells. **a** Cell cycle analysis upon CDC6 induction and plot of re-replicating cells (DNA content > 4n). **b** Comet assay showed DNA breaks in cells induced for the indicated time points. DNA damage was significantly reduced in the “escaped” cells. Plot depicts tail comet (moment) calculations. Red lines depict moment tails. Scale bar: 35 μm. **c** DDR activation in CDC6-induced cells as demonstrated by double IF for 53BP1 and CDC6 (Scale bar: 20 μm) along with western blotting for p53 and p21^WAF1/Cip1^. DDR is reduced in the “escaped” cells. Actin serves as a loading control.

**Fig. 4:**
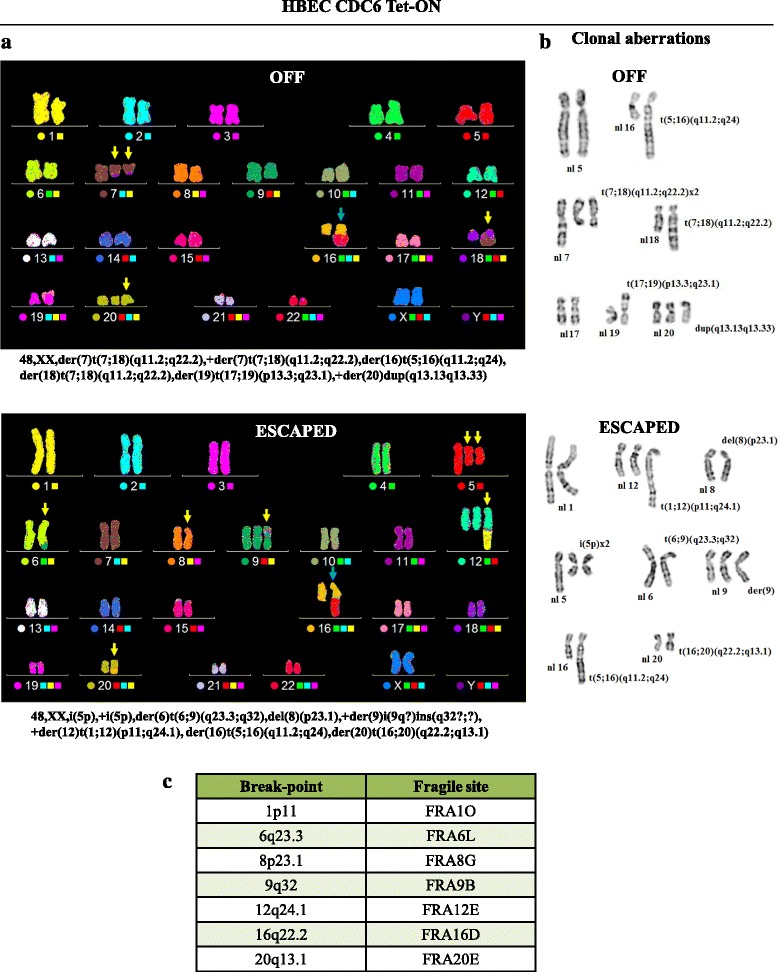
Cytogenetic analysis revealed an altered karyotype in the “escaped” cells, displaying novel clonal numerical and structural chromosome aberrations coinciding with common fragile sites (CFS). **a** Multi-Color FISH spectral karyotyping (M-FISH/SKY) of the non-induced and the “escaped” HBECs combined with inverted DAPI banding, as described in [143], revealed a near diploid numerical constitution of 48 chromosomes. Yellow arrows indicate unique clonal rearrangements for each population whereas the blue arrows depict the common structural anomaly der(16)t(5;16). **b** Partial karyotypes in Inverted DAPI Banding demonstrate the breakpoints of clonal structural chromosome rearrangements in the two populations, OFF (upper panel) and “escaped” (lower panel). nl: normal copy. Several clonal structural rearrangements characteristic of the control cells (OFF), were lost in the cytogenetically examined “escaped” cells; these included the products of the translocation t(7;18), der(7)t(7;18)(q11.2;q22.2)(found in two copies) and der(18)t(7;18)(q11.2;q22.2). Lost in the “escaped” cells were also the der(19)t(17;19)(p13.3;q23.1) and the extra copy of chromosome 20 bearing a duplication of the long arm. Despite the clonal chromosome losses, the “escaped” cells displayed at least 6 novel clonal structural chromosome anomalies and their cytogenetic constitution was shaped by non-disjunctions of chromosomes 1, 5, 7, 12 16, 18 and 19. The karyotype of the "escaped" cells was characterized by the clonal gain of genomic material from 1q and most of chromosome 12, introduced by the emergence of the novel unbalanced translocation involving chromosomes 1 and 12, der(12)t(1;12)(p11;q24.1), as well as deletions of 8p23.1. In addition to these novel anomalies, the “escaped” cells presented an unbalanced translocation involving chromosomes 6 and 9, an extra copy of a rearranged derivative of chromosome 9, a translocation between 16 and 20 and two copies of isochromosome 5p. **c** Breakpoints of all the novel structural chromosome rearrangements identified in the “escaped” HBECs coincide with common aphidicolin induced fragile sites [78]

In line with our previous reports [33, 60, 75], CDC6 induction resulted in re-replication (cells with DNA content > 4n) (Fig. 3a), a form of replication stress, that leads to replication fork stalling, collapse, DNA damage and DDR activation. DNA damage was documented by alkaline comet assay (Fig. 3b), and DDR stimulation by 53BP1 foci formation and induction of the p53 pathway (Fig. 3c). The emergence of the “escaped” cells was distinguished, apart from the EMT features, by the attenuation of the DNA damage and the DDR pathway, implying that a repair process took place. A cytogenetic analysis of the “escaped” cells, relative to the non-induced (OFF) ones, revealed the presence of an altered karyotype, implying that this repair process was error-prone. The "escaped" cells harbored novel clonal (numerical and structural) chromosomal alterations and an increased rate of random structural chromosome rearrangements, indicative of genomic instability (Fig. 4a-b and Additional file 8: Figure S5) [76, 77]. Of note, we have observed a similar molecular response in cells that “escaped” from protracted p21^WAF/Cip1^-mediated senescence [64]. Remarkably, the majority of the novel breakpoints identified in the “escaped” HBECs coincided with aphidicolin-induced CFS (Fig. 4c) [78], a finding that is in line with the proposed oncogene-induced DNA damge model for cancer development [36]. Interestingly, the karyotype of the “escaped” cells was also characterized by gains of 1q and 5p and deletions of 8p23.1, which are frequently found in common solid cancers and various hematologic malignancies, and are associated with poor prognosis [79–82].

Even though replication stress via re-replication can explain CDC6-mediated genomic instability, a recent report showing that CDC6 could regulate *rDNA* transcription initiation [66] led us to presume that an additional source of genomic instability could be R loop formation. R loops are three-stranded nucleic acid structures that encompass nascent RNA hybridized with DNA template, leaving single-stranded the non-template DNA (ssDNA) [83] (Additional file 9: Figure S6). Their formation and/or stabilization, which follows transcription, depends on various factors such as, high G density, negative supercoiling, DNA nicks and G-quartets in the displaced ssDNA [84, 85], and if persistent they set genome integrity at “risk” [83]. They are also frequently produced at CFS, regions of the genome prone to replication stress, located in long human genes (≥ 800 kb); thus increasing the possibility of replication-transcription collision and genomic instability [56]. The fact that R loops are reported in vivo at origins of replication [86–90] and *rDNA* loci [91, 92] increases the probability of their formation by deregulated expression of the replication licensing factor, CDC6.

**Fig. 5:**
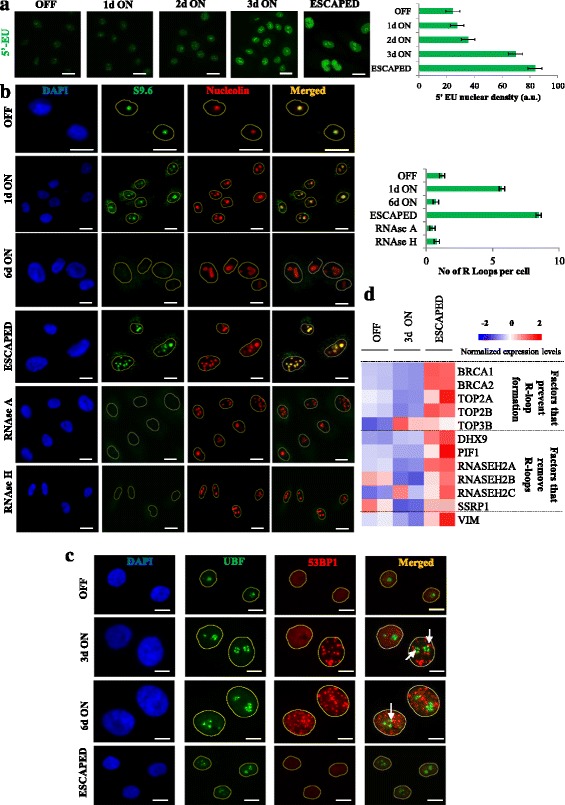
CDC6 induction resulted in R loop formation. **a** CDC6 induction increased total transcription levels, as measured by 5’-EU incorporation. Scale bar: 35 μm. **b** IF for S9.6, antibody specific for DNA:RNA hybrids, indicated increased possibility of R loop formation upon CDC6 expression. Concurrent IF detection of nucleolin, revealed nucleolus subcellular localization of the R loops. DNA:RNA hybrids disappeared after treatment with RNase A or RNase H showing the specificity of the reaction. Scale bar: 15 μm. **c** Double IF for 53BP1 and UBF showed re-localization of UBF from the nucleolar interior to the periphery associated with the perinucleolar distribution of 53BP1 foci (indicated by white arrows) reflecting DNA damage repair in heterochromatin-related structures [96]. Scale bar: 10 μm. **d** Heatmap of factors affecting R loops (Additional file 11: Table S4) [98]

To examine the above scenario we first measured total transcription levels by quantifying 5′-ethynyl uridine (5’-EU) incorporation at specific time points, pre- and post-CDC6 induction. We observed a gradual increase of 5’-EU integration that peaked in the “escaped” cells (Fig. 5a). Re-replication and RNA production could well form a permissive genome landscape for the generation of R loops. Indeed, applying a specific antibody that detects DNA:RNA hybrids (S9.6) revealed a raise in R loop formation within the nucleoli in the initial phases of CDC6 expression that ceased during senescence, possibly due to recession of DNA replication, but reappeared in the “escaped” cells (Fig. 5b). The specificity of the reaction was verified by treating the cells with RNase A or RNase H, enzymes specific in removing DNA:RNA hybrids [93, 94] (Fig. 5b). If R loops were related to DNA damage in our setting, then a repositioning of damaged rDNA on the nucleolar surface should occur facilitating recruitment of repair factors. This type of peripheral re-localization of the repair process is characteristic of heterochromatin enriched structures, such as the nucleoli [95, 96]. In agreement with the latter, induction of CDC6 in HBECs resulted in the re-localization of UBF (upstream binding factor) signifying rDNA [97], from nucleolar interior to the periphery, where it is associated with 53BP1 foci, forming nucleolar caps (Fig. 5c) [96].

It has been suggested that R loops may demonstrate dissimilar structural features depending on their role within the cell. They could be characterized as “Janus-face” modules with beneficial or adverse functions; the nature of which has not been linked with particular structural traits, yet [98]. Within this context, vimentin expression (Fig. 2c), a cardinal trait of EMT, in the “escaped” cells could be R loop-dependent, as previously reported [99]. Transcriptome analysis of the “escaped” cells revealed elevated expression of factors that prevent or remove R loops, whereas most of them were reduced during the initial phases of CDC6 induction (Fig. 5d, Additional file 10: Figure S7 and Additional file 11: Table S4). The differential expression of these R loop-affecting factors in the “escaped” cells compared to early stage CDC6-induced ones may be viewed as an adaptation to the high demand for protein-synthesis of the “escaped”-protumorigenic cells; thus reducing the risk for replication-transcription collisions at the *rDNA* loci possibly favoring the beneficial effects of the R loops.

**Fig. 6:**
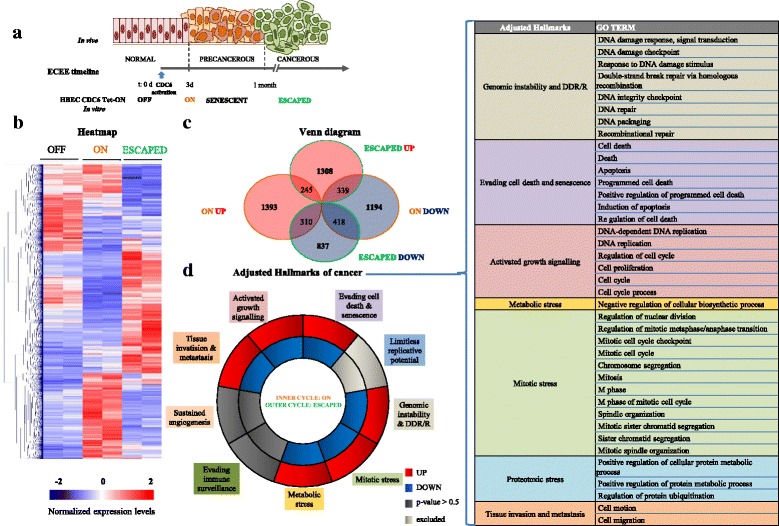
Transcriptome analysis of HBEC CDC6 Tet-ON cellular system. **a** Timeline of ECEE (epithelial cancer evolution experiment) showing time points where main biochemical and phenotypical events occur. High-throughput RNA sequencing analysis that was performed on 3-day induced (initiation of senescence phase) and in the “escaped” cells, compared to non-induced ones, revealed extensive alterations in the transciptome landscape. **b** Heatmap, showing hierarchical clustering, and (**c**) Venn diagram of the deregulated genes indicated that most of them were exclusive features of either of the two time points and not common ones nominating that they share different traits. **d** Adjustment of the transcriptome analysis to the “hallmarks” of cancer, utilizing Gene Ontology (GO) terms as shown in table, revealed that the “escaped” cells share the characteristic features of cancer cells. DDR/R refers to DNA damage response and repair pathways

In an attempt to reveal functional modules related to cancer initiation and progression we adjusted the data from the transcriptome and the cytogenetic analyses (Figs. 4 and 6) to the “hallmarks” of cancer [4, 73, 100]. As depicted in Fig. 6 the results further strengthened the value of HBEC CDC6 Tet-ON cellular system to study “escape from senescence” and carcinogenesis, as the “escaped” cells share most of the cancerous characteristics, adopting a stress phenotype [101]. Of note, transcriptomics showed different transcriptome landscapes affecting major biological processes in the senescent versus the "escaped" cells (Fig. 6d). Moreover, gene-set enrichment analysis demonstrated that the cell cycle-associated pathway and DNA replication were essentially stopped in the induced cells and significantly up-regulated once the cells escaped from senescence (Additional file 12: Figure S8). In the analysis, we did not include “limitless replicative potential”, as ectopic expression of hTERT could lead to artificially positive results. Notably, the only “hallmarks” that could not be scored as statistically significant were those of “evading immune surveillance” and “sustained angiogenesis”, probably due to the lack of surrounding stroma.

**Fig. 7:**
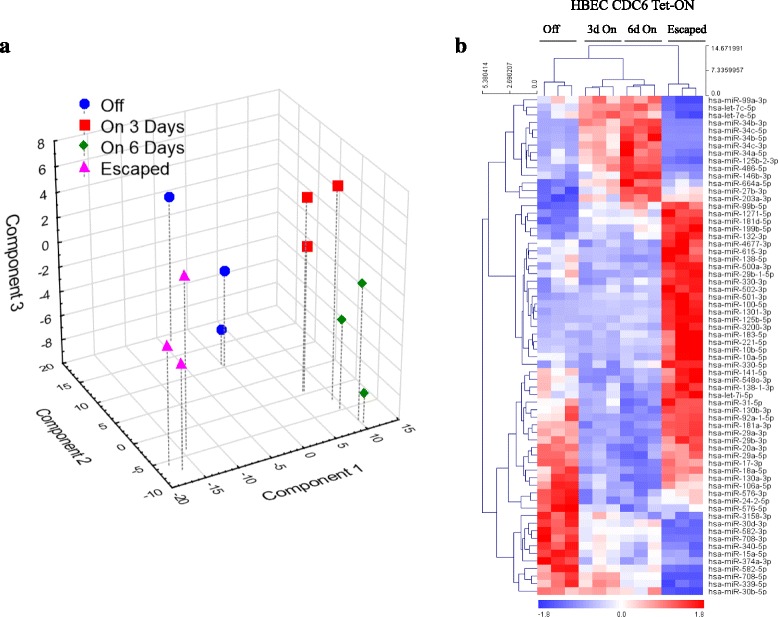
MiR analysis of HBEC CDC6 Tet-ON cellular system. **a** Principal component analysis of miR profiles showing that after escaping from the senescence programme, the cells reverted to the pre-induction stage, but considerable alterations of their miR expression persisted. **b** Hierarchical clustering of miRs that showed significant differences between the non-induced (OFF), induced (3d On and 6d On) and escaped from senescence (Escaped) cells

Global miR expression analysis (miRseq) (Additional file 13: Table S5) yielded similar to RNAseq results, showing significantly different profiles between the senescent and "escaped" populations (Fig. 7). Two miRs were of particular interest: miR34c and miR29a. The first one showed significant up-regulation in the CDC6 over-expressing cells at day 3, followed by a further rise at day 6, whereas in the "escaped" cells, its levels fell below those observed in the non-induced cells, verified by qRT-PCR analysis (Additional file 14: Figure S9). According to reports, miR34c along with a panel of additional miRs up-regulated at day 6, are related to senescence (Additional file 15: Table S6). On the other hand, miR29a showed an inverse pattern. Of note, miR29a has been shown to play a role in EMT [102]. Interestingly, emerging data support that down-regulation of *miR34* genes, including *miR34c*, are also implicated in EMT through a negative feedback loop with SNAIL, which comes in line with our findings [103]. Over-represented pathways, gene ontologies and target genes are summarized in Additional file 16: Table S7.

### *In situ* co-detection of gene coding (protein) and non-coding (miR) products during OIS

As mentioned, the role of miRs in senescence is well established [19, 20, 22]. However, the technical limitations of the SA-β-Gal method (see Background section), has rendered miR *in situ* detection in senescent cells unfeasible so far. The advantages of the GL13 reagent and the described features of the HBEC CDC6 Tet-ON system (induction of senescence, escape from senescence and activation of the DDR-p53 pathway) prompted us to explore this model for monitoring, in situ, the spatiotemporal expression pattern of a gene coding (protein) and non-coding (miR) product functionally linked with the processes of DDR and senescence. Prompted by the miRseq analysis we selected miR34c as a miR target. MiR34a-c are amongst the best-characterized direct transcriptional downstream targets of p53, placing them as bona fide components of the p53 network [104]. In addition, miR-34s, including miR34c, were reported to trigger senescence in various human lung settings [104, 105]. On the other hand, 53BP1 is an ideal protein target since it is a well-established upstream mediator of the DDR pathway, which is recruited to sites of double strand breaks (DSBs) forming discrete foci [55, 106–108]. For *in situ* co-detection we followed a three-step immuno-fluorescence process: **i)** Fluorescence *in situ* hybridization (FISH) for miR34c, followed by **ii)** GL13 staining to spot senescent cells, and finally **iii)** detection of 53BP1 foci. Successful GL13 staining and 53BP1 foci formation (steps ii and iii) were independently confirmed during the validation of the HBEC CDC6 Tet-ON system, as shown in Figs. 1d and 3c and Additional file 7: Figure S4b, respectively.

**Fig. 8:**
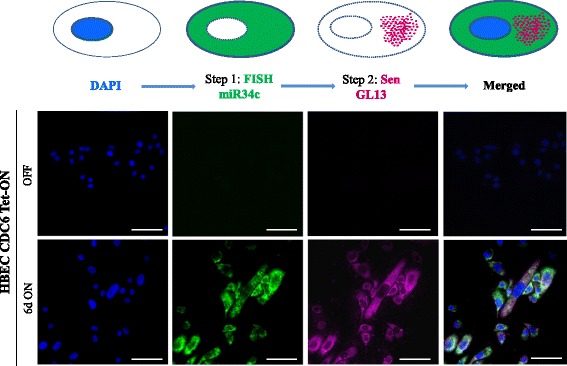
*In situ* detection of miR34c in senescent cells. Detection of miR34c in senescent cells employing the HBEC CDC6 Tet-ON system. Double staining was performed in two consecutive states: in the OFF state where proliferation of HBECs is evident (“OFF”) and 6 days after constitutive induction of CDC6 when cells are senescent (“6d ON”). Step 1: Fluorescence FISH of miR34c employing a double-DIG-labeled LNA probe, visualized as green emission in the cytoplasm, using TSA plus Fluorescein (emitting at 518 nm). Step 2: GL13 staining, visualized at far red spectra as granules in the cytoplasm employing Alexa Fluor goat-anti-mouse (647 nm) (emitting at 668 nm). Scale bar: 50 μm

**Fig. 9:**
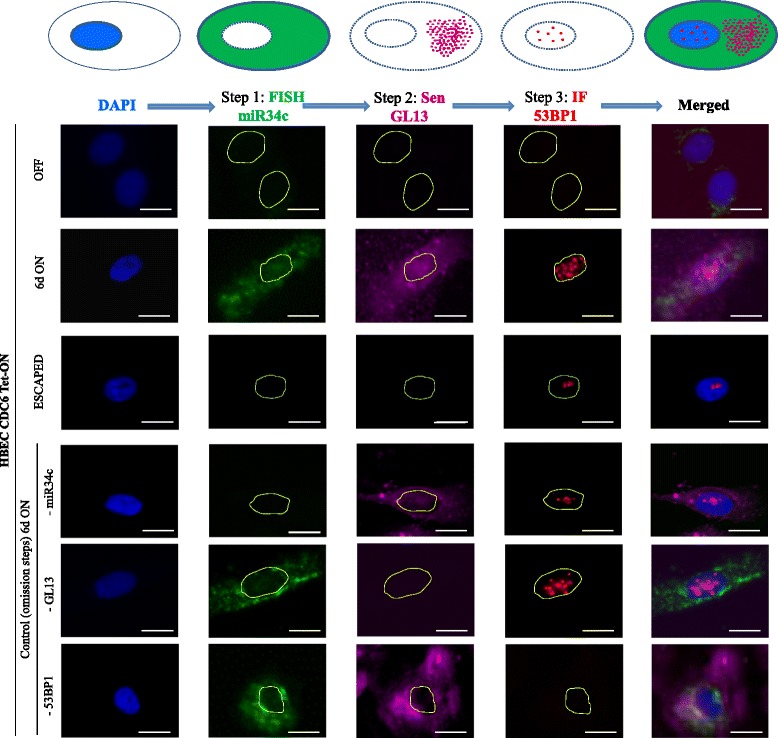
Monitoring concurrently *in situ* miRs and proteins expression during OIS. Co-detection of miR34c and 53BP1 in senescent cells, employing the HBEC CDC6 Tet-ON system and our proposed protocol (see Additional file 17 and Additional file 18: Figure S10). Multiple staining was performed in three consecutive states: in the OFF state where proliferation of HBECs is evident (“OFF”), 6 days after constitutive induction of CDC6 when cells are senescent (“6d ON”) and in the “escape from senescence” state termed “ESCAPED”. Step 1: miR34c FISH employing a double-DIG-labeled LNA probe, visualized as green emission in the cytoplasm, using TSA plus Fluorescein (emission at 518 nm). Step 2: GL13 staining, visualized at far red spectra as granules in the cytoplasm employing Alexa Fluor goat-anti-mouse (647 nm) (emission at 668 nm). Step 3: 53BP1 IF, visualized as red foci in the nucleus, employing Alexa Fluor goat-anti-mouse (568 nm) (emission at 618 nm). The specificity of each individual probe/antibody was tested by omitting sequentially the following reagents: miR34c probe, GL13 and anti-53BP1 antibodies at 6d ON cells. U6 snRNA and Scramble FISH, serving as positive and negative controls respectively, are presented in the Additional file 20: Figure S12. Scale bar: 20 μm

Due to the challenging nature of the 3-step co-detection procedure, detailed technical aspects, control and trouble-shooting processes are described meticulously in the Additional file 17 and illustrated in Additional file 18: Figure S10. Only the principles of the *in situ* assay are presented and discussed in the current section. **Step 1; miR34c detection:** Before proceeding with ISH of miR34c, its expression following CDC6 induction was confirmed by qRT-PCR (Additional file 14: Figure S9). For ISH we employed the Locked Nucleic Acid (LNA) technology [109–111]. LNAs are nucleic acid analogues “locked” by a methylene bridge that constrains them in the ideal conformation for Watson-Crick binding allowing superior hybridization properties [112] (Additional file 19: Figure S11). To increase detection sensitivity we used double-digoxigenin (DIG) labeled (at both 5′ and 3′ ends) probes visualized with the Tyramide Signal Amplification (TSA) Plus Fluorescein System emitting at 517 nm [113–115]. Although the water-soluble 1-ethyl-3-(3-dimethylaminopropyl) carbodiimide (EDC) increases signal strength, we omitted it since it interferes with protein detection [116]. As negative and positive controls of the method we employed Double-DIG labeled Scramble-miR and U6 small nuclear RNA (snRNA) probes, respectively (Additional file 20: Figure S12). **Step 2; Detection of senescent cells**: To detect miR34c positive senescent cells we employed the GL13 reagent [53] that overcomes the restrictions of the SA-β-Gal assay (see Background section) allowing examination of the sensitive to decay miR molecules to precede senescence detection. **Step 3; Detection of 53BP1 foci:** Finally, we proceeded with 53BP1 foci detection using conventional immunofluorescent (IF) analysis. Each secondary antibody emitted at different wave-lengths to avoid overlapping during visualization (see Methods section, Figs. 8 and 9).

As depicted in Figs. 8 and 9 following a two (steps 1 and 2) and a three step (steps 1, 2 and 3) process we successfully co-detected miR34c in senescent cells that clearly showed evidence of DDR activation (53BP1 foci). For each step a parallel experiment took place, omitting the primary reagent (miR34c probe, GL13 and anti-53BP1 antibody), to exclude false positive staining from the secondary antibodies. Notably, miR34c was not detected in the “escaped” cells (Fig. 9), which is in agreement with the miRseq and qRT-PCR analysis (Additional file 14: Figure S9), probably because of declined p53 levels in these cells (Fig. 3c).

## Discussion

A major challenge in understanding the pathogenesis of cancer is the availability of models that functionally recapitulate observations from human clinical settings. A successful model addresses the following questions: How relevant is it to human cancer? What type of human cancer does it mimic? Is the recapitulated event an initiating or a promoting one? How can it help uncover or predict mechanism(s) that could be therapeutically targeted?

While in vivo mice models are the first choice, their development is costly and time consuming. Moreover, their relevance to human carcinogenesis has been questioned despite the large body of knowledge gained till now [117]. In mice the quantity of genetic events required for cellular transformation is lower compared to humans [100, 118], whereas qualitative alterations differ, as well. As an example, in humans activated oncogenes are encountered mainly by the DDR pathway, while in mice activation of the p19ARF tumor-suppressor predominates [58, 59, 119–121]. Additionally, telomeres in humans are shorter than in mice, possibly as an anti-tumor protective mechanism to eliminate cells that have acquired dangerous mutations during the relative long human lifespan [122].

Human cell lines represent established alternatives as they are more relevant to human disease, although there are certain limitations, such as the absence of complex intercellular and cell-matrix interactions that exist in in vivo models [100]. Still, they facilitate the direct interrogation of cancer associated genetic events, like oncogene activation, and their impact on cellular outcomes, such as evasion from the anti-tumor barriers of apoptosis and senescence [36].

In this study, we developed and present an original human non-malignant epithelial (bronchial) oncogene-inducible cellular system that recapitulates the precancerous and cancerous phases of epithelial carcinogenesis precisely, within a relatively short time frame (Figs. 1 and 6a). Non-malignant epithelial platforms are generally rare as most researchers prefer fibroblast- or cancer cell line-based settings because they are manipulated much easier. However, the cellular context determines to a great extent the carcinogenetic process. Given that the majority of cancers are of epithelial origin, non-malignant epithelial systems are essential to study the initiating events of epithelial carcinogenesis. Prompted by the fact that our model showed a potent senescent response, following oncogene activation, we took advantage of GL13, a novel senescence biomarker we synthesized, and monitored concurrently *in situ* miR and protein expression, for the first time, during OIS. The latter was not feasible until now due to inherited flaws of SA-β-Gal, the currently available method [40], whereas GL13 enables detection of senescent cells in any given biological material [53].

As a triggering oncogenic stimulus we favored the replication licensing factor CDC6 mainly because it is frequently over-expressed in various epithelial cancers from their earliest stages of development [60, 61]. Moreover, we previously showed that its deranged expression is not a mere reflection of increased proliferation but a potential “driving force”, as suggested by the oncogene-induced replication stress model for cancer development, we proposed few years ago [33, 36, 62]. Lying in the heart of the replication machinery we anticipated that CDC6 induction in the HBEC setting would mimic from an evolutionary perspective the precancerous and cancerous stages of epithelial cancer development.

Indeed, CDC6 over-expression provoked a rapid senescent response that was maintained for a prolonged period (precancerous phase) (Fig. 1 and Additional file 7: Figure S4). In parallel, at the molecular level an intense DDR response was noticed, which is in line with previous reports demonstrating a functional link between DDR and acquirement of senescence [33, 34, 64, 72, 74]. The most interesting finding appeared at day 30 post-induction when cellular clones with phenotypical and molecular traits of EMT emerged (“escaped” cells - cancerous phase) (Figs. 1d and 2c and Additional file 7: Figure S4). EMT is an embryonic program that confers to cancer invasion and progression when reactivated at the “wrong time” [70, 71]. The DDR was alleviated in the “escaped” cells implying that an extensive repair process occurred. Judging from the novel clonal and random chromosomal alterations observed in the “escaped” cells (Fig. 4 and Additional file 8: Figure S5), an error-prone repair procedure probably took place, the exact nature of which remains to be elucidated. A similar process has been recently described during chronic p21^WAF1/Cip1^ expression in a p53-independent environment [64]. In addition to the EMT features, the “escaped” cells possessed most of the hallmarks of cancer, as depicted from the transcriptome analysis of the “escaped” versus the non-induced cells (Fig. 6d), clearly pinpointing the value of our cellular system to study induction and escape from senescence.

Apparently and in agreement with the oncogene-induced replication stress model for cancer development, CDC6-driven genomic instability exerted a selective pressure that led to the evolution of the “escaped” cells. The fact that this process was raised during the senescent phase puts forward a scenario according to which senescence should not be viewed as a “static” state, but as a “dynamic” one, when the genome landscape is continuously reformed and shaped. Although re-replication is “incriminated” as the means by which CDC6 causes DNA damage, the current study unraveled that R loops could represent and additional source of CDC6 mediated genomic instability [83]. R loop formation occurred within the nucleoli, heterochromatin nuclear substructures comprised by the *rDNA* region. The *rDNA* locus is a highly repetitive region of the genome consisting of tandem repeats encoding the rRNA subunits and is enriched with origins of replication. The latter along with the fact that CDC6 was shown to activate the transcription of *rDNA* [66] renders its loci prone to R loop formation, replication-transcription collision and DNA damage. In support to this notion, we observed in CDC6-induced HBECs redistribution of UBF to the periphery of the nucleoli localized adjacent to 53BP1 foci, forming the so-called nucleolar caps (Fig. 5c) [96]. Having in mind that UBF is the main transcription factor of *rDNA* [123] and repair of heterochromatin DNA takes place at its periphery [95, 124], we can deduce that R-loop formation in our setting most probably led to DNA damage; thus contributing to CDC6-driven genomic instability.

Conclusively, we believe that the experimental system we present can inspire further mechanistic studies and address questions such as which origins of replication deregulated CDC6 activates. Are they intergenic or intrangenic? In the latter case what is the risk of replication-transcription collision? What type of error-prone repair pathway(s) ensues during CDC6 induced senescence? Are the transcriptional properties of deranged CDC6 restricted only to the *INK4/ARF*, *CDH1* (E-cadherin) and *rDNA* loci or does CDC6 mediate a global transcriptional program? If yes, does it involve displacement of the chromosomal insulator CTCF as it does for the *INK4/ARF* and *CDH1* loci? Moreover, with regard to the miRseq analysis, which of the detected miRs are directly involved in inducing senescence and which are implicated in the escape phenomenon? All the above questions and their answers gain particular worth within the context of the non-malignant epithelial environment of our system as they most probably signify cancer initiating events.

## Conclusions

The desire to simultaneously detect multiple intracellular macromolecules has its roots back to late 60’s [125]. Despite progress in the development of *in situ* detection assays during the last decades, multiple *in situ* staining still remains a challenge. This is pertinent to the field of senescence because of the additional practical limitations exerted by the application of the SA-β-Gal assay. Prompted by this widespread need, we have developed and described herein a methodology that bypasses these restrictions. We have provided proof-of-principle that this methodology maintains optimal preservation of the biological setting and tremendous flexibility, enabling gold standard ISH techniques to be combined with antigen detection and senescence marker recognition.

To validate the new method, we have developed a cell system based on human bronchial epithelial cells that recapitulates the whole spectrum of epithelial carcinogenesis following the inducible over-expression of the replication licensing factor CDC6. The vast majority of in vitro models used to study processes related to malignant transformation are fibroblast-based, which contrast the fact that most malignancies are of epithelial origin. Thus, the HBEC CDC6 Tet-ON epithelial system emerges as a valuable prototypical tool that possesses the following advantages: **i)** It enables phenotypic and molecular monitoring of OIS and escape from senescence, recapitulating the precancerous and cancerous phases of human epithelial carcinogenesis. In the future it could be applied in co-cultures with stromal cells; hence investigating the interplay between the various phases of OIS and the surrounding cellular environment. **ii)** The properties of CDC6 acting concurrently as a replication and transcription factor, when deregulated, will facilitate the in-depth study of genome dynamics, such as re-replication, replication-transcription collision, DNA repair in conjunction with transcriptional deregulation, and chromatin remodeling. Along these lines we have observed temporal formation of R loops that cease during senescence and reappear upon escape. **iii)** Applying the senescence detecting reagent GL13, which lacks the limitations of the SA-β-Gal assay, provides the unique opportunity to monitor co-currently *in situ* both proteins and regulatory RNAs, such as miRs. Moreover, the utilization of the GL13 compound for detection of lipofuscin is applicable not only to track senescence, but also to monitor escape from it. The latter is due to the fact that lipofuscin is non-degradable, accumulating progressively and is diluted only through cell divisions. As a result, traces of lipofuscin in the cells escaping from senescence can be detected rendering it a unique lineage tracing marker [126]. We expect that this cell model, coupled with the multifaceted applications that the new macromolecule detection methodology offers, will enable a more direct examination of the spatiotemporal expression of miRs and gene-coding products relevant to senescence and malignant transformation.

## Methods

### Cell culture, plasmids and HBEC CDC6 Tet-ON system generation

The Lenti-X™ Tet-On® 3G Inducible Expression System (Clontech Laboratories) was employed to establish a CDC6 inducible-expression cellular model in immortalized HBECs (hTERT/CDK4) [57], which were a kind gift of Dr. T. Liloglou.

PLVX-TRE3G-CDC6 was generated by digesting pTRE2Hyg-CDC6 [63] with BamHI and EcoRV (blunt end) and subcloning MYC-tag-hCDC6-cDNA into PLVX-TRE3G linearized with BamHI and SmaI (blunt ended). Immortalized HBECs are resistant to G418 due to the neomycin-resistant gene introduced with the CDK4 expression vector (pSRα-MSU) and to puromycin due to the corresponding-resistant gene introduced with p-babe-hygro-hTERT. Given that PLVX-TET3G and PLVX3G-TRE-CDC6 carry also neomycin-resistant and puromycin-resistant genes, accordingly, it was necessary to replace the resistance cassettes of lentiviral vectors with blasticidin-resistant (BSD) and zeocin-resistant (BleoR) genes. Particularly, IRES-BSD from pBIB was transferred into pBluescript SK with EcoRI–ClaI and then obtained with KpnI and BamHI to replace IRES-Neo-WPRE from PLVX-TET3G using partial digestion. BleoR was derived from Lenti X1 zeo-pTERshATM with XbaI-filled ends and KpnI to replace puromycin-restistant cassette of PLVX3G-TRE-CDC6 obtained with MluI-filled ends and KpnI. For a schematic presentation of vectors see Fig. 1a.

Production of lentiviruses and transduction were performed according to supplier’s guidelines utilizing Lenti-X™ Concetrator (Clontech Laboratories). After two-week selection with 3 μg/mL blasticidin and 12.5 μg/mL zeocin, cell clones with doxycyclin-dependent (1 μg/mL, Sigma) induction of CDC6-MYC-tagged, as assessed by immunoblot and real-time qRT-PCR analyses (Fig. 1a), were isolated and used for the described experiments. Upon CDC6 induction, doxycyclin was replenished every second day.

Immortalized HBECs and HBEC CDC6 Tet-ON cells were maintained in Keratinocyte Serum-Free Medium (#17005–075, Invitrogen) supplemented with 50 μg/ml Bovine Pituitary Extract and 5 ng/ml hEGF (#17005–075, Invitrogen) at 37 °C and 5% CO_2_ [57].

Microphotographs were obtained with an inverted microscope (Axiovert S100; Carl Zeiss) equipped with CP-Achromat objectives and a charge-coupled device IRIS colour video camera (SSC-C370P; Sony), using Image Pro Plus v3.0 (Media Cybernetics) software.

### Total protein extraction and western blot analysis

Total protein extracts were obtained by homogenization in 50 mM Tris/HCl pH 8.0, 150 mM NaCl, 0,1% SDS, 0,5% sodium deoxycholate, 1% NP-40 adjusted with protease and phosphatase inhibitors. The homogenate was centrifuged at 13,400 rpm at 4 °C for 10 min. The supernatant was collected and protein content quantified using Protein assay dye concentrate (BIO-RAD). Thirty μg of protein from total extracts, were adjusted with Laemmli Buffer (Sigma) and loaded on acrylamide/bis-acrylamide gels. Gel electrophoresis, transfer to PVDF membrane (Millipore) and signal development with chemiluminescence substrate ECL for HRP (PerkinElmer) were performed as previously described [60]. Primary antibodies were used at the following dilutions: CDC6 (Santa Cruz #9964) 1:1000, p-RB (Santa Cruz #7986-R) 1:500, RB (Santa Cruz #50) 1:1000, p53 (Santa Cruz #47698) 1:1000, p21^WAF1/Cip1^ (Santa Cruz #6246, 1:400), Actin (Cell Signaling #4967, 1:1000). Anti-mouse (Cell Signaling #7076) and anti-rabbit (Cell Signaling #7074) HRP-linked secondary antibodies diluted at 1:1000 were used.

### RNA isolation, cDNA preparation and qRT-PCR

RNA was extracted using Nucleospin RNA (Macherey-Nagel #740955) according to the manufacturer’s instructions. 1 μg RNA was used for cDNA preparation with Primescript™ RT Reagent Kit (Takara #RR037A). Real-time qRT-PCR analysis was performed utilizing SYBR Select Master Mix (Life technologies #4472908) on a DNA-Engine-Opticon (MJ-Research) thermal cycler. Primer sequences are as follows, CDC6 forward: 5’-CAGTTCAATTCTGTGCCCGC-3′ and reverse: 5’-GCTCCTTCTTGGCTCAAGGT-3′, β2-microglobulin (reference gene) primers were forward: 5’-TCGCGCTACTCTCTCTTTCT-3′ and reverse: 5’-TTTCCATTCTCTGCTGGATGAC-3′. Results, averaged from three independent experiments, are presented as n-fold changes for the various time points after CDC6 induction versus the values of the non-induced sample, using the 2-ΔΔ^CT^ method.

For miR detection, RNA was extracted with the NucleoSpin miR, kit/50preps (Cat no: 740971.50; Macherey-Nagel), following the manufacturer’s instructions. For reverse transcription the TaqMan® MicroRNA RT Kit, 200 RXNS (Cat no: 4366596, ThermoFischer Scientific) along with TaqMan® Universal Master Mix II with UNG (Cat no: 4440042, ThermoFischer Scientific) and the two TaqMan® MicroRNA Assays Inv sM10 (Cat no: 4427975, ThermoFisher Scientific) were employed. The assays ID for each of the corresponding primers and probes are the following: a) hsa-miR-34c (target assay): 000428 and b) U6snRNA (control assay): 001973. Expression of miR-34c was calculated relative to U6snRNA levels according to the comparative method of 2-^ΔΔCT^. Relative quantification of miR expression was calculated from three independent biological replicates.

### ChIP assay

ChIP assay was performed in HBEC CDC6 Tet-ON cells grown in 100 mm plates and induced for 2 days. Cells were cross-linked with 1% formaldehyde in 50 mM Hepes-KOH pH 7.5, 100 mM NaCl, 1 mM EDTA pH 8.0, 0.5 mM EGTA pH 8.0 for 15 min at RT on a rocking platform. Cross-linking was stopped by the addition of glycine to a final concentration of 125 mM for 5 min at RT on a rocking platform. Cross-linked cells were washed twice with ice cold PBS, scraped and centrifuged at 3000 rpm for 5 min. Approximately 6 × 10^6^ cells were resuspended in 2 ml of lysis buffer (50 mM Hepes pH 7.9, 140 mM NaCl, 1 mM EDTA, 10% glycerol, 0.5% NP-40, 0.25% Triton-X100) and incubated for 10 min on ice in the presence of protease and phosphatase inhibitors. Cells were then washed twice in 10 mM Tris-HCl pH 8.1, 200 mM NaCl, 1 mM EDTA pH 8.0, 0.5 mM EGTA pH 8.0, two more times in shearing buffer (0,1% SDS, 10 mM Tris-HCl pH 8.1, 1 mM EDTA pH 8.0) and resuspended in 1 ml of shearing buffer in the presence of protease and phosphatase inhibitors. Cells were sonicated with a Covaris S2 sonicator for 15 min. Triton-X100 and NaCl was added to a final concentration of 1% and 150 mM, respectively. The debris was pelleted by 10-min centrifugation at 13000 rpm at 4 °C, and the soluble chromatin material was precleared with salmon sperm DNA/50% protein A agarose slurry. Further steps were performed as described before [63].

### BrdU proliferation assay

For BrdU incorporation, cells grown on coverslips were pulse-labeled with 10 μM BrdU (Roche) overnight at 37 °C. Cells with incorporated BrdU were treated and visualized by indirect IF analysis as described in the corresponding methodology subsection.

### Senescence/GL13 staining

GL13 staining was performed as described before [53]. GL13 compound is commercially available as SenTraGor™ from Arriani Pharmaceuticals (Cat no: AR8850040).

### SA-β-gal assay

SA-β-Gal activity was detected according to *Debacq-Chainiaux* et al. [127].

### Wound healing assay

Cells were seeded on 100 mm tissue-culture plastic dishes at 70% confluence and, the next day, a scratch wound was performed using a sterile 200 μl pipette tip. Phase-contrast images were taken at the starting (0 h) time point and at 8 h and 24 h time intervals using an inverted microscope (Axiovert S100; Carl Zeiss).

### Electron microscopy analysis

Cells were fixed in a freshly-prepared solution containing 3% formaldehyde (prepared from paraformaldehyde) and 0.5% glutaraldehyde in 0.1 M phosphate buffer, pH 7.4, for 30 min at room temperature (RT). Cells were then harvested using a scraper, collected into a tube and centrifuged at 800 g for 5 min at RT. The supernatant was aspirated, while cells were resuspended in 4% gelatin warmed aquatic solution followed by a spin down at 800 g for 5 min at RT and cooled on ice. Under a stereoscope the solidified cell pellet with gelatin was extracted, cut into small fragments (1–2 mm^3^) and transferred into 0.1 M phosphate buffer, pH 7.4 at 4 °C. The cell-gelatin fragments were then dehydrated in graded series of ethyl alcohol, followed by propylene oxide (PO) treatment, infiltrated gradually in a mixture of Epon/Araldite resins diluted in PO and finally embedded in fresh epoxy resin mixture. Ultrathin epoxy sections (70-90 nm thickness) were cut on a Leica Ultracut R ultramicrotome, equipped with a Diatome diamond knife, and mounted onto 200-mesh copper grids. Ultrathin sections were observed with a Philips 420 transmission electron microscope and micrographs were taken with an Olympus Megaview G2 CCD camera.

### Indirect IF analysis

For indirect IF analysis cells were grown on coverslips and fixed with 100% ice-cold methanol or 4% formaldehyde (prepared from paraformaldehyde) for 10 min and store at 4 °C until staining was performed. Following, cells were permeabilized with 0,3% Triton X-100 in PBS for 5 min at RT. A 10% fetal bovine serum and 3% bovine serum albumin in PBS solution was used as a blocking buffer for 1 h at RT. Primary antibodies were diluted in blocking buffer and incubated overnight at 4 °C. Secondary antibodies were goat anti-rabbit or goat anti-mouse, Alexa Fluor® 488 or Alexa Fluor® 568 (Invitrogen) diluted 1:500 in blocking buffer. Counterstaining was performed with 100 ng/ml DAPI (Sigma-Aldrich). Primary antibodies used were: CDC6 (Santa Cruz #9964, 1:100 or #8341, 1:50), Cyclin A (Santa Cruz #239, 1:150), E-cadherin (Cell Signaling #3195 1:200), Vimentin (Dako M0720, 1:50) 53BP1 (abcam #21083, 1:250), S9.6 (Kerafast ENH001, 1:200), nucleolin (Cell Signaling #14574, 1:1000) and UBF (Santa Cruz #13125, 1:100). For S9.6 IF, treatment with RNase A (0.25 μg/μl) or RNase H (50 units/slide) at 37 °C for 30 min and 3 h, respectively, was performed if necessary. Image acquisition from multiple random fields was automatically obtained on a ScanR screening station (Olympus, Germany) and analyzed with ScanR (Olympus, Germany) software, or a Zeiss Axiolab fluorescence microscope equipped with a Zeiss Axiocam MRm camera and Achroplan objectives, while image acquisition was performed with AxioVision software 4.7.1.

### Flow cytometric analysis (FACS)

Cell cycle analysis was assessed on a FACS Calibur (Becton-Dickinson) as previously described [63].

### Comet assay

Comet assay was performed with minor modifications of a standard previously described protocol [63]. In brief, after trypsinization and wash in PBS, 200,000 viable cells were resuspended in 225 μl of TBE (Tris–Boric acid–EDTA) buffer, mixed with 1275 μl of low melting agarose embedded in plugs. Plugs were subsequently incubated in 10 ml lysis solution (100 mM Tris-HCl, 100 mM EDTA, and 2.5 M NaCl, pH 10, with the addition of 1.25% Triton X-100 and 10% DMSO, before use) overnight on ice and in the dark. After completion of lysis, plugs were washed twice in TBE for 30 min on ice in the dark. Finally, plugs were washed and subsequently incubated in ice-cold alkaline denaturation buffer (300 mM NaOH and 1 mM EDTA, pH 13) for 45 min on ice in the dark. For electrophoresis, plugs were mounted onto 1% agarose-coated slides that were placed into a 30-cm horizontal constant-field gel electrophoresis chamber in ice-cold alkaline denaturation buffer for 30 min at 0.7 V/cm and at 4 °C. After electrophoresis, slides were washed 3 times in TBE, dehydrated in ice-cold ethanol (100%) for 15 min and then allowed to dry in the dark. 24 h later, slides were rehydrated in 5 ml of deionized water for 10 min, and 40 μl of diluted SYBR gold (Invitrogen, Molecular Probes) was applied on each plug. Cells were observed under a fluorescence microscope (Axiolab) equipped with a monochrome UV camera (XC-EU50 CE; Sony). Analysis was conducted using the CometScore software (TriTek Corp.).

### 5’-EU incorporation based nascent RNA assay

*In situ* detection of nascent RNA was performed with the Click-iT Alexa Fluor 488 Imaging Kit (Invitrogen, Molecular Probes). Briefly, cells were incubated for 30 min in the presence of 0.5 mM 5-EU. Samples were fixed in 4% formaldehyde for 15 min and permeabilized in 0.5% Triton X-100 for 20 min at RT. Samples were then processed according to the manufacturer’s recommendation. Cells were analyzed using LSM780 or LSM710 (Carl Zeiss Microscopy) confocal microscopes and 5-EU nuclear intensity was quantified with the NIS-elements software (Nikon).

### Molecular Cytogenetics

Molecular cytogenetics analysis was conducted as previously published [63]. Specifically, a 63× magnification lens on a fluorescent Axio-Imager Z1, Zeiss microscope equipped with a MetaSystems charge-coupled device camera and the MetaSystems Isis software were used.

### RNA-sequencing (RNAseq) and miR-sequencing (miRseq) preparation and analysis

The library preparation for RNAseq and miRseq was carried out in the Greek Genome Center (GGC) of Biomedical Research Foundation of Academy of Athens (BRFAA). RNA was collected from biological duplicates of HBEC CDC6 Tet-ON non-induced (OFF), 3d ON and “escaped” cells. RNAseq libraries were prepared with the TruSeq RNA kit using 1 μg of total RNA. The libraries were constructed according to Illumina’s protocols and then were mixed in equal amounts. Paired-end 38 bp reads for 2 “OFF”, 2 “ON” and 2 “ESCAPED” status samples were generated with NextSeq500 in the GGC. RNAseq raw sequencing data were aligned to human genome version GCCh37/hg19 with the use of tophat (version 2.0.9) [128] and the use of «--b2-very-sensitive» parameter. Data filtering and file format conversion was performed with Samtools (version 0.1.19) [129]. Aligned reads were assigned into exons using HT-seq count (version 0.6.1p1) algorithm [130] with the following command «htseq-count –s no –m intersection -nonempty». Finally differentially expressed genes were identified with the use of DESeq R package [131] and genes with at least 10 reads, fold change cut off 1.5 and *p*-value ≤0.05 were considered to be differentially expressed (DEGs) (Additional file 11: Table S4).

For miRseq the procedure was carried out differently. After automatic filtering, adapter trimming, error removing and quality control using AfterQC [132], good quality reads were mapped directly to miRbase (release 21) database of all mature miR sequences using Novocraft’s NovoAlign tool. This allowed us to create raw count tables accounting for every present miR. Raw counts were then normalized using standard transcripts per million (tpm) normalization. Length of miRs was acquired from miRbase v21. Four groups were compared: non-induced cells (OFF), induced ones (ON at 3 and 6 days) and "escaped" from senescence (ESC). MiRs present in at least 10 transcripts per million in at least three samples from any of the groups were selected for comparisons, yielding the total number of 269 unique miRs ready for analysis. The four groups were compared with one-way ANOVA. MiRs with Bonferroni-adjusted *p* < 0.05 were entered into a hierarchical clustering and principal component analyses (Additional file 13: Table S5). Post-hoc comparisons between the groups were performed using a Tukey’s test.

#### Bioinformatic and functional analyses

DAVID knowledgebase [133] was used for Gene Ontology analysis. Only pathways and biological processes with p-value ≤0.05 (Fisher’s exact test) were considered to be significantly enriched. RNAseq data have been deposited in the Short Read Archive (SRA) under the accession codes PRJNA388146. Gene set enrichment analysis (GSEA) [134] was performed using the canonical pathways (Cp2) subset of the molecular signatures database [http://software.broadinstitute.org/gsea/msigdb/].

For miRs, the over-representation analysis was performed using miRNA enrichment analysis miEAA [135]. Over-represented pathways and gene ontologies were searched using the miRWalk and the search for target genes utilized the miRTarBase as reference. To account for multiple comparison problems we lowered the threshold for statistical significance to 0.001 as it was impossible to fully adjust for multiple hypothesis testing and the number of between group analyses. Additionally, the threshold of at least 3 miRs present in the analyzed sets was applied. MiRseq data have been deposited in GEO with accession number GSE106588.

## Additional files

Additional file 1: Figure S1. Biogenesis pathway and subcellular localization of miRs. miRs are mainly transcribed by RNA pol II, while a cluster of miRs flanked by Alu repeats on chromosome 19 (C19MC) are transcribed by RNA pol III [136], into pri-miRs (>1kb long) with a hairpin structure [137]. Pri-miRs are recognized by Drosha, a class 2 RNAase III, and an RNA binding protein (RBP) called DGCR8/Pasha. Drosha cleaves the 5’ and 3’ arm of the hairpin releasing pre-miRs (~70bp long). The latter are exported through the nuclear pores into the cytoplasm by Exportin 5 in association with Ran-GTP. In the cytoplasm processing of pre-miRs is mediated by Dicer, a class 3 RNase III, which along with various RBPs, including TRBP, stabilize Dicer. Dicer-TRBP complex liberates small RNA duplexes that are loaded onto Argonaute protein members (Ago1-4) forming effector complexes called pre-RISCs. Pre-RISCs remove the passenger miRs strand generating the mature form of RISCs encompassing single strand miRs (~22 nucleotides long each). The functional strand of miRs loaded on Ago1-4 guides RISCs to silence target mRNAs in the cytoplasm (C) through translational repression, mRNA cleavage and deadenylation [138]. Additionally, miRs may translocate into: a) the nucleus (N) [16], regulating the biogenesis of coding and non-coding RNAs (active RISC complexes are present in the nucleus (nRISC) having a distinct composition from cytoplasmic RISC (cRISC) [139]) and b) the mitochondria (M) bound to Ago2 at pre-RISC or mature RISC complex (mRISC) [17], regulating the translation of the mRNAs produced by mitochondrial genome which, in turn, modulate mitochondrial homeostasis [140]. Evidence also supports the presence of mitochondrial miRs encoded by mitochondrial genome [18]. A substantial fraction of miRs may also exist in the cytoplasm in an Ago-free form [141]. Notably, apart from DGCR8 and TRBP, different RBPs recognize distinct miR precursors regulating miR biogenesis [142]. (PDF 1025 kb)

Additional file 2: Table S1. Subcellular localization of miRs. (XLSX 12 kb)

Additional file 3: Table S2. Studies on miR expression in senescent cells. (XLSX 20 kb)

Additional file 4: Table S3. Genes triggering oncogene-induced senescence. (XLSX 12 kb)

Additional file 5: Figure S2. RB phosphorylation in HBEC CDC6 Tet-ON system. Immunoblot analysis of total and phosphorylated RB levels. CDK4 over-expression in HBEC results in continuous phorsphorylation of RB protein, while induction of CDC6 increased p-RB due to transcriptional down-regulation of p16 [63]. Actin serves as loading control. (PDF 21 kb)

Additional file 6: Figure S3. CDC6 binding onto the promoters of *CDH1* and *INK4/ARF* loci of HBEC CDC6 Tet-ON system leading to transcriptional down-regulation. a) Chromatin immunoprecipitation (ChIP) assay showed that MYC-tagged CDC6 is bound on both the regulatory domain (RD) of *INK4/ARF* locus and the Epal element of *CDH1*, when induced. b) RD of *INK4* locus is enriched in DNA extracted from both anti-CDC6 (endogenous and exogenous) and anti-MYC-tag (exogenous) IPs in HBEC CDC6 over-expressing cells normalized to input and *INK4b intron* (RNA Pol II-IP serves as a negative control confirming transcriptional down-regulation). c) ChIP samples run on a SDS-PAGE gel revealed that CDC6 is accessible and immunoprecipitated by both CDC6 and MYC-tag antibodies with the protocol followed. (PDF 146 kb)

Additional file 7: Figure S4. Morphological features of HBEC CDC6 Tet-ON. a) Inverted-phase contrast photographs (Scale bar: 25 μm) and bi) GL13 staining showed the dominance of senescent, flattened and multinucleated cells upon 6-day CDC6-induction; features that were substituted by a spindle morphology in the “escaped” cells. Traces of GL13 staining in the early "escaped" cells (indicated by arrows) prove their origin from senescent cells. bii) Sa-β-Gal activity correlates with GL13 staining. (Scale bar: 15 μm). (PDF 689 kb)

Additional file 8: Figure S5. Comparative Inverted DAPI Banding karyotyping of 20 metaphase spreads from the OFF (on the left) and the “escaped” (on the right) cells. Arrows indicate random chromosome rearrangements (chromosomal instability). The rates of random structural chromosome rearrangements were found 3.5-times more pronounced in the "escaped" cells. (PDF 554 kb)

Additional file 9: Figure S6. Schematic presentation of an R loop. R loops are three-stranded nucleic acid structure. Factors (upper left corner) that promote R loops are indicated as well as the differential cellular effects (bottom) stemming from their formation. (PDF 557 kb)

Additional file 10: Figure S7. Bedgraphs of indicative genes showing the specificity of RNAseq analysis. RNAseq data from two biological replicates is depicted. (PDF 47 kb)

Additional file 11: Table S4. A Upregulated genes in induced (ON) HBEC CDC6 Tet-ON cells. b Down regulated genes in induced (ON) HBEC CDC6 Tet-ON cells. c Upregulated genes in ESCAPED (ESC) HBEC CDC6 Tet-ON cells. d: Down regulated genes in ESCAPED (ESC) HBEC CDC6 Tet-ON cells. (XLSX 630 kb)

Additional file 12: Figure S8. Enrichment plots a-b) of the “Cell cycle mitotic” and c-d) of the “DNA replication” gene-sets. Cells entering senescence (3-day induced) showed a significant (Bonferroni-adjusted *p* value <0.001) down-regulation of cell-cycle and DNA replication pathways in comparison to control ones. These changes were reversed with a significant up-regulation (Bonferroni-adjusted *p* value <0.001) of both sets when the cells escaped from senescence. (PDF 528 kb)

Additional file 13: Table S5. A MiR expression differences between induced (3d ON) and non-induced (OFF) HBEC CDC6 Tet-ON cells. FWER - Bonferroni-adjusted p value. b MiR expression differences between induced (6d ON) and non-induced (OFF) HBEC CDC6 Tet-ON cells. FWER - Bonferroni-adjusted p value. c MiR expression differences between "escaped" (ESC) and non-induced (OFF) HBEC CDC6 Tet-ON cells. FWER - Bonferroni-adjusted p value. d MiR expression differences between 3-day induced (3d ON) and 6-day induced (6d ON) HBEC CDC6 Tet-ON cells. FWER - Bonferroni-adjusted p value. e MiR expression differences between "escaped" (ESC) and induced (3d ON) HBEC CDC6 Tet-ON cells. FWER - Bonferroni-adjusted p value. f MiR expression differences between "escaped" (ESC) and induced (6d ON) HBEC CDC6 Tet-ON cells. FWER - Bonferroni-adjusted p value. (XLSX 134 kb)

Additional file 14: Figure S9. miR-34c expression analysis in OFF, ON and “ESCAPED” HBEC CDC6 Tet-ON cells utilizing: a) qRT-PCR and b) miRseq analysis, tpm (transcripts per million). (PDF 25 kb)

Additional file 15: Table S6. Potential miR signature of senescence according to miRseq analysis of HBEC CDC6 Tet-ON system and existing literature. (XLSX 9 kb)

Additional file 16: Table S7. MiR enrichment analysis showing pathways, Gene Ontology terms and Target genes significantly linked to differentially-expressed miRs between respective groups.Fields marked out in bold represent pathways present in at least two pairwise comparisons. (XLSX 20 kb)

Additional file 17: Supplementary methods. (see also Additional file 18: Figure S10). (DOC 91 kb)

Additional file 18: Figure S10. Flowchart of the protocol employed to co-detect *in situ* gene coding (protein) and non-coding (miR) products during OIS in the HBECs CDC6 Tet-ON system. See also Additional file 17. (PDF 116 kb)

Additional file 19: Figure S11. Hybridization probes incorporating nucleotide analogs based on the LNA technology. (PDF 144 kb)

Additional file 20: Figure S12. Detection of U6 snRNA and scramble-miR miRNACURY control double-DIG labeled probes employing TSA plus Fluorescein system. Scale bar: 30 μm. (PDF 74 kb)

## Abbreviations

5’-EU: 5′-ethynyl uridine
BrdU: Bromodeoxyuridine
CDC6: Cell division cycle 6
CDK4: Cyclin-dependent kinase 4
CFS: Common fragile sites
ChIP: Chromatin immunoprecipitation,
DDR: DNA damage response
DGCR8: DiGeorgi critical region 8
DIG: Double-digoxigenin
DSB: Double strand break
ECEE: Epithelial cancer evolution experiment
EDC: 1-ethyl-3-(3-dimethylaminopropyl) carbodiimide
EMT: Epithelial to mesenchymal transition
FACS: Flow cytometric analysis
FISH: Fluorescence *in situ* hybridization
GO: Gene ontology
HBEC: Human bronchial epithelial cells
hTERT: Human telomerase reverse transcriptase
IF: Immunofluorescence
ISH: *in situ* hybridization
LNA: Locked nucleic acid
M-FISH/SKY: Multicolour fluorescence *in situ* hybridization/spectral karyotypicing
miR: Micro-RNA
ncRNA: Non-coding RNA
OIS: Oncogene-induced senescence
Pasha: Partner of Drosha
PO: Propylene oxide
pre-miR: Precursor micro-RNA
pri-miR: Primary micro-RNA
qRT-PCR: Quantitative reverse-transcription polymerase chain reaction
RBP: RNA binding protein
RD: Regulatory domain
*rDNA*: Ribosomal DNA
RISC: RNA-induced silencing complex
RNA pol: RNA polymerase
RNAseq: RNA sequencing
RS: Replicative senescence
RT: Room temperature
SAHF: Senescence associated heterochromatin foci
SASP: Senescence associated secretory phenotype
SA-β-Gal: Senescence-associated β-galactosidase
SIPS: Stress-induced premature senescence
snRNA: Small nuclear RNA
ssDNA: Single-stranded
TRBP: TAR RNA-binding protein
TSA: Tyramide signal amplification
UBF: Upstream binding factor
