# Correction to: A prototypical non-malignant epithelial model to study genome dynamics and concurrently monitor micro-RNAs and proteins in situ during oncogene-induced senescence

**DOI:** 10.1186/s12864-021-07608-z

**Published:** 2021-05-05

**Authors:** Eirini-Stavroula Komseli, Ioannis S. Pateras, Thorbjørn Krejsgaard, Konrad Stawiski, Sophia V. Rizou, Alexander Polyzos, Fani-Marlen Roumelioti, Maria Chiourea, Ioanna Mourkioti, Eleni Paparouna, Christos P. Zampetidis, Sentiljana Gumeni, Ioannis P. Trougakos, Dafni-Eleftheria Pefani, Eric O’Neill, Sarantis Gagos, Aristides G. Eliopoulos, Wojciech Fendler, Dipanjan Chowdhury, Jiri Bartek, Vassilis G. Gorgoulis

**Affiliations:** 1 Molecular Carcinogenesis Group, Department of Histology and Embryology, School of Medicine, National & Kapodistrian University of Athens, 75 Mikras Asias St, GR-11527 Athens, Greece; 2 Department of Immunology and Microbiology, University of Copenhagen, Blegdamsvej 3c, DK-2200 Copenhagen, Denmark; 3 Department of Biostatistics and Translational Medicine, Medical University of Lodz, 15 Mazowiecka St., 92-215 Lodz, Poland; 4 Biomedical Research Foundation of the Academy of Athens, 4 Soranou Ephessiou St., GR-11527 Athens, Greece; 5 Department of Cell Biology and Biophysics, Faculty of Biology, National & Kapodistrian University of Athens, GR-15784 Athens, Greece; 6 CRUK/MRC Institute for Radiation Oncology, Department of Oncology, University of Oxford, Oxford, OX3 7DQ UK; 7 Department of Biology, School of Medicine, National & Kapodistrian University of Athens, 75 Mikras Asias St., GR-11527 Athens, Greece; 8 Institute of Molecular Biology and Biotechnology, Foundation for Research & Technology-Hellas, GR-70013 Heraklion, Crete Greece; 9 Department of Radiation Oncology, Dana-Farber Cancer Institute, 450 Brookline Ave, Boston, MA 02215 USA; 10 Harvard Medical School, 25 Shattuck St, Boston, MA 02115 USA; 11 Genome Integrity Unit, Danish Cancer Society Research Centre, Strandboulevarden 49, DK-2100 Copenhagen, Denmark; 12 Institute of Molecular and Translational Medicine, Faculty of Medicine and Dentistry, Palacky University, Hněvotínská, 1333/5, 779 00 Olomouc, Czech Republic; 13 Department of Medical Biochemistry and Biophysics, Karolinska Institute, Science for Life Laboratory, Division of Translational Medicine and Chemical Biology, SE-171 77 Stockholm, Sweden; 14 Faculty of Biology, Medicine and Health, University of Manchester, Manchester Academic Health Science Centre, Wilmslow Road, Manchester, M20 4QL UK

**Correction to: BMC Genomics 19, 37 (2018)**

**https://doi.org/10.1186/s12864-017-4375-1**

Following publication of the original article [1], it was reported that earlier versions of Table S4a and Table S4d in Additional file 11 were uploaded. The correct version of Additional file 11 has been included here, and the original article has been corrected.

## Supplementary Information

**Additional file 11: Table S4a.** Upregulated genes in induced (ON) HBEC CDC6 Tet-ON cells. **Table S4b.** Down regulated genes in induced (ON) HBEC CDC6 Tet-ON cells. **Table S4c.** Upregulated genes in ESCAPED (ESC) HBEC CDC6 Tet-ON cells. **Table S4d.** Down regulated genes in ESCAPED (ESC) HBEC CDC6 Tet-ON cells.
