# Supplementary figures and images for: A prototypical non-malignant epithelial model to study genome dynamics and concurrently monitor micro-RNAs and proteins in situ during oncogene-induced senescence
Source: BMC Genomics. 2018 Jan 10;19:37. doi: 10.1186/s12864-017-4375-1 (PMC5763532; doi:10.1186/s12864-017-4375-1)

Figure S1

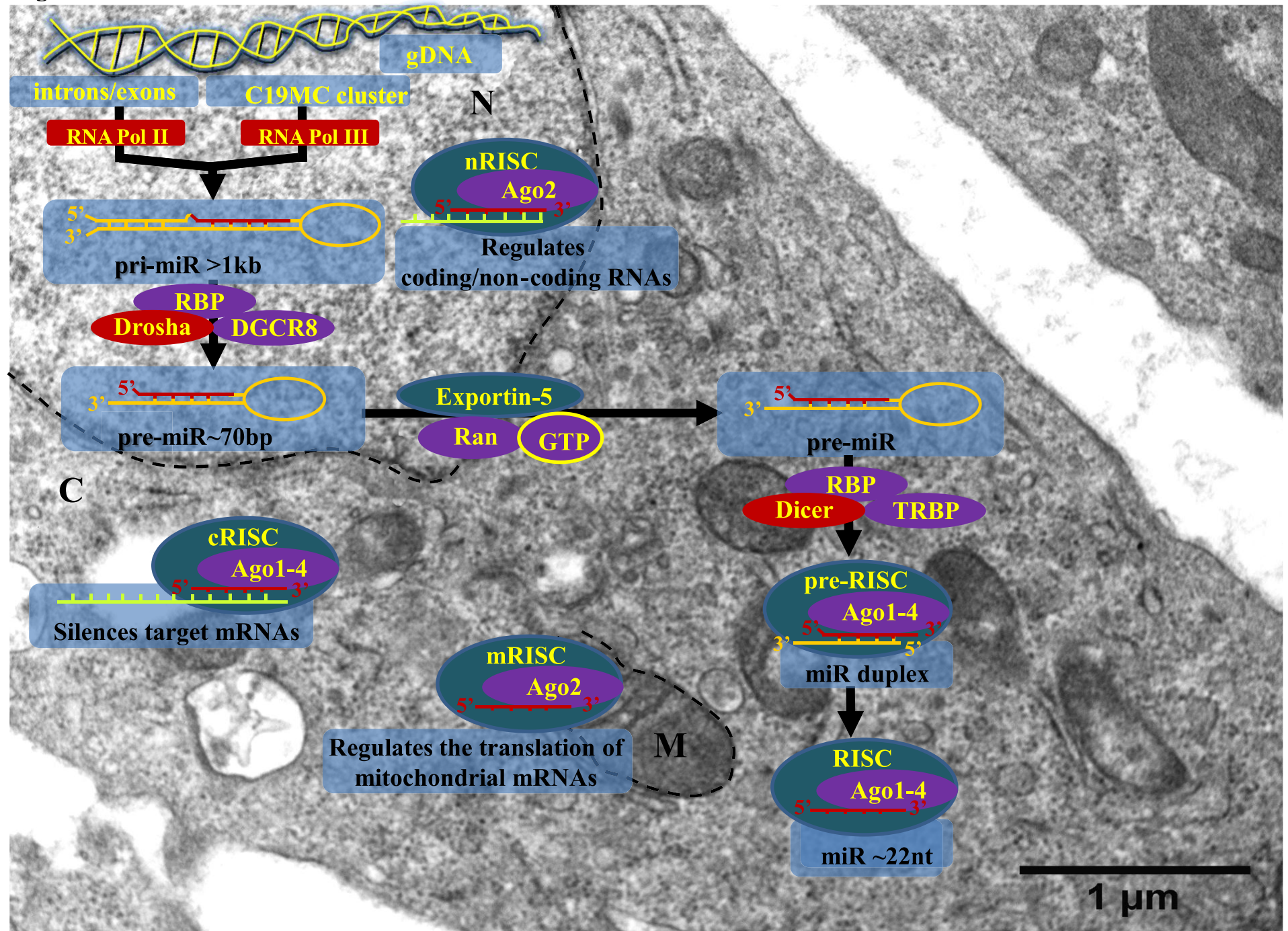

Supplement: Supplementary file 1 — Biogenesis pathway and subcellular localization of miRs. miRs are mainly transcribed by RNA pol II, while a cluster of miRs flanked by Alu repeats on chromosome 19 (C19MC) are transcribed by RNA pol III [136], into pri-miRs (>1kb long) with a hairpin structure [137]. Pri-miRs are recognized by Drosha, a class 2 RNAase III, and an RNA binding protein (RBP) called DGCR8/Pasha. Drosha cleaves the 5’ and 3’ arm of the hairpin releasing pre-miRs (~70bp long). The latter are exported through the nuclear pores into the cytoplasm by Exportin 5 in association with Ran-GTP. In the cytoplasm processing of pre-miRs is mediated by Dicer, a class 3 RNase III, which along with various RBPs, including TRBP, stabilize Dicer. Dicer-TRBP complex liberates small RNA duplexes that are loaded onto Argonaute protein members (Ago1-4) forming effector complexes called pre-RISCs. Pre-RISCs remove the passenger miRs strand generating the mature form of RISCs encompassing single strand miRs (~22 nucleotides long each). The functional strand of miRs loaded on Ago1-4 guides RISCs to silence target mRNAs in the cytoplasm (C) through translational repression, mRNA cleavage and deadenylation [138]. Additionally, miRs may translocate into: a) the nucleus (N) [16], regulating the biogenesis of coding and non-coding RNAs (active RISC complexes are present in the nucleus (nRISC) having a distinct composition from cytoplasmic RISC (cRISC) [139]) and b) the mitochondria (M) bound to Ago2 at pre-RISC or mature RISC complex (mRISC) [17], regulating the translation of the mRNAs produced by mitochondrial genome which, in turn, modulate mitochondrial homeostasis [140]. Evidence also supports the presence of mitochondrial miRs encoded by mitochondrial genome [18]. A substantial fraction of miRs may also exist in the cytoplasm in an Ago-free form [141]. Notably, apart from DGCR8 and TRBP, different RBPs recognize distinct miR precursors regulating miR biogenesis [142]. (PDF 1025 kb) [file 12864_2017_4375_MOESM1_ESM.pdf]

**Figure S2**

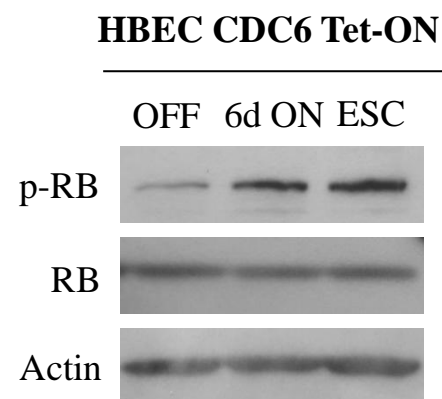

Supplement: Supplementary file 5 — RB phosphorylation in HBEC CDC6 Tet-ON system. Immunoblot analysis of total and phosphorylated RB levels. CDK4 over-expression in HBEC results in continuous phorsphorylation of RB protein, while induction of CDC6 increased p-RB due to transcriptional down-regulation of p16 [63]. Actin serves as loading control. (PDF 21 kb) [file 12864_2017_4375_MOESM5_ESM.pdf]

### Figure S3

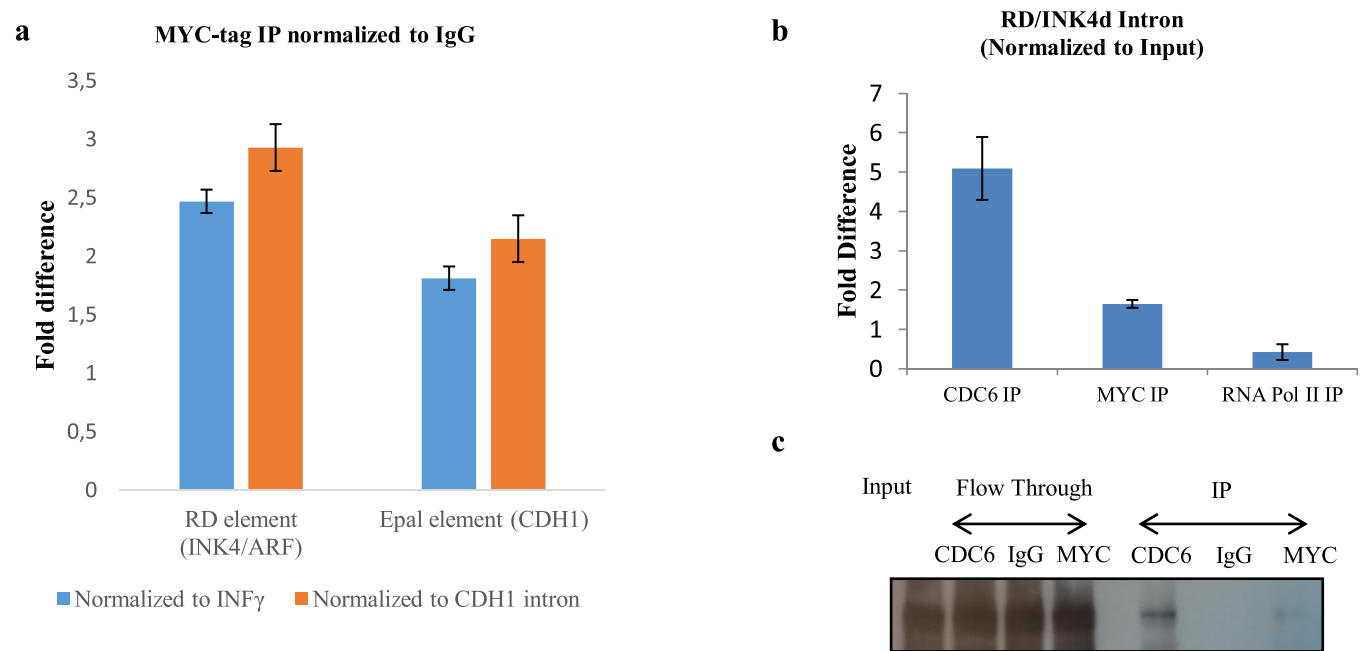

Supplement: Supplementary file 6 — CDC6 binding onto the promoters of CDH1 and INK4/ARF loci of HBEC CDC6 Tet-ON system leading to transcriptional down-regulation. a) Chromatin immunoprecipitation (ChIP) assay showed that MYC-tagged CDC6 is bound on both the regulatory domain (RD) of INK4/ARF locus and the Epal element of CDH1, when induced. b) RD of INK4 locus is enriched in DNA extracted from both anti-CDC6 (endogenous and exogenous) and anti-MYC-tag (exogenous) IPs in HBEC CDC6 over-expressing cells normalized to input and INK4b intron (RNA Pol II-IP serves as a negative control confirming transcriptional down-regulation). c) ChIP samples run on a SDS-PAGE gel revealed that CDC6 is accessible and immunoprecipitated by both CDC6 and MYC-tag antibodies with the protocol followed. (PDF 146 kb) [file 12864_2017_4375_MOESM6_ESM.pdf]

Figure S4

a. HBEC CDC6 Tet-ON (phase contrast)

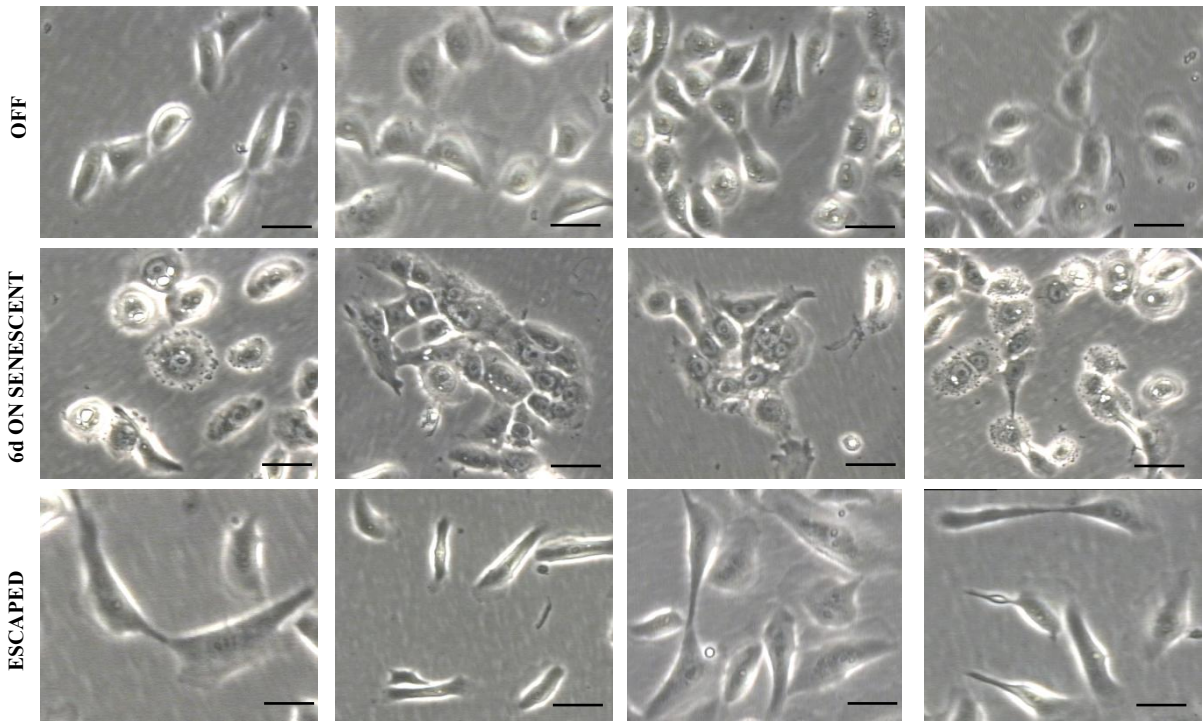

bi.

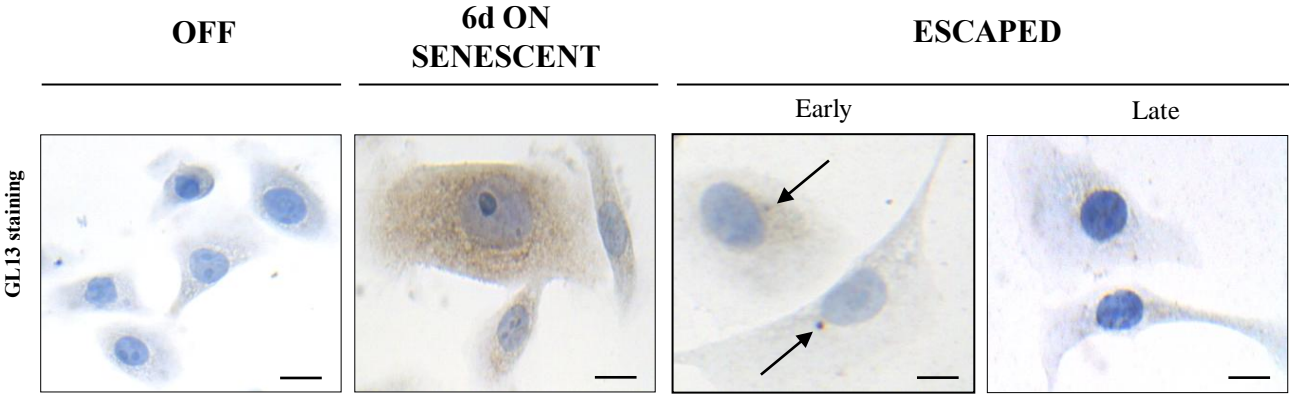

bii.

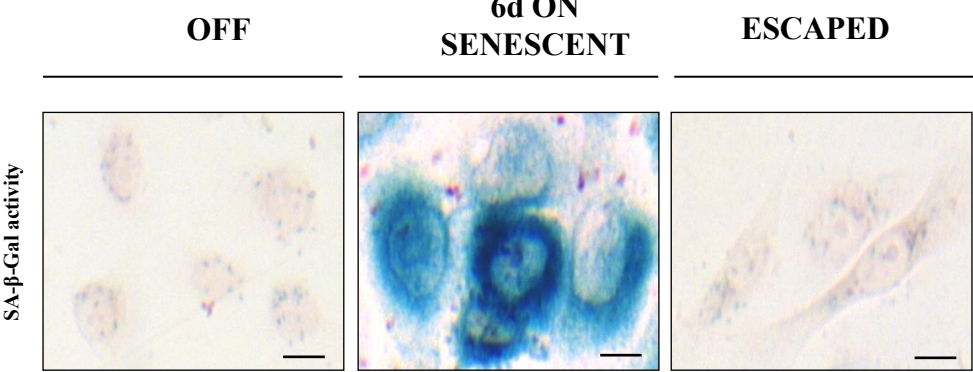

Supplement: Supplementary file 7 — Morphological features of HBEC CDC6 Tet-ON. a) Inverted-phase contrast photographs (Scale bar: 25 μm) and bi) GL13 staining showed the dominance of senescent, flattened and multinucleated cells upon 6-day CDC6-induction; features that were substituted by a spindle morphology in the “escaped” cells. Traces of GL13 staining in the early "escaped" cells (indicated by arrows) prove their origin from senescent cells. bii) Sa-β-Gal activity correlates with GL13 staining. (Scale bar: 15 μm). (PDF 689 kb) [file 12864_2017_4375_MOESM7_ESM.pdf]

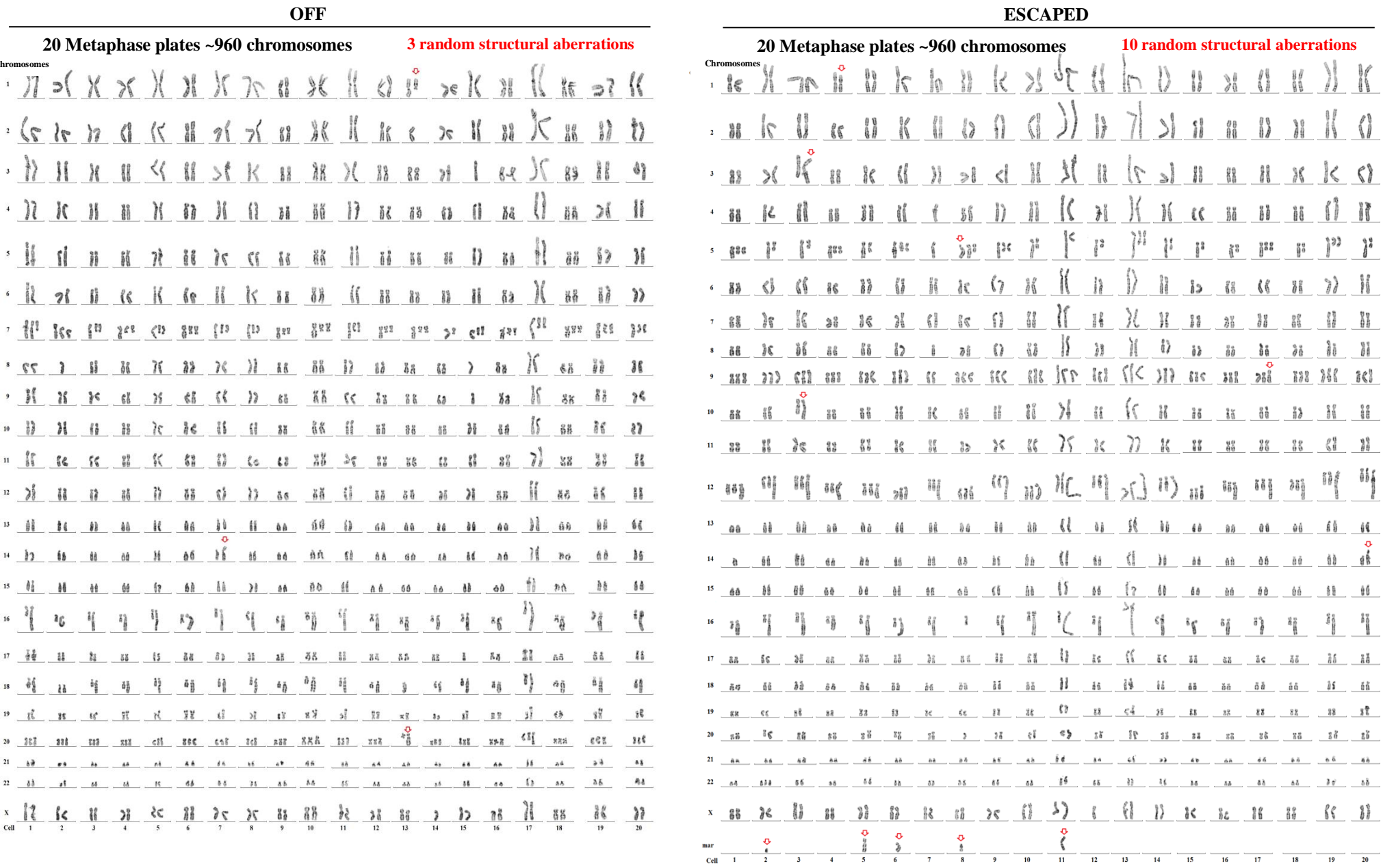

Supplement: Supplementary file 8 — Comparative Inverted DAPI Banding karyotyping of 20 metaphase spreads from the OFF (on the left) and the “escaped” (on the right) cells. Arrows indicate random chromosome rearrangements (chromosomal instability). The rates of random structural chromosome rearrangements were found 3.5-times more pronounced in the "escaped" cells. (PDF 554 kb) [file 12864_2017_4375_MOESM8_ESM.pdf]

Figure S6

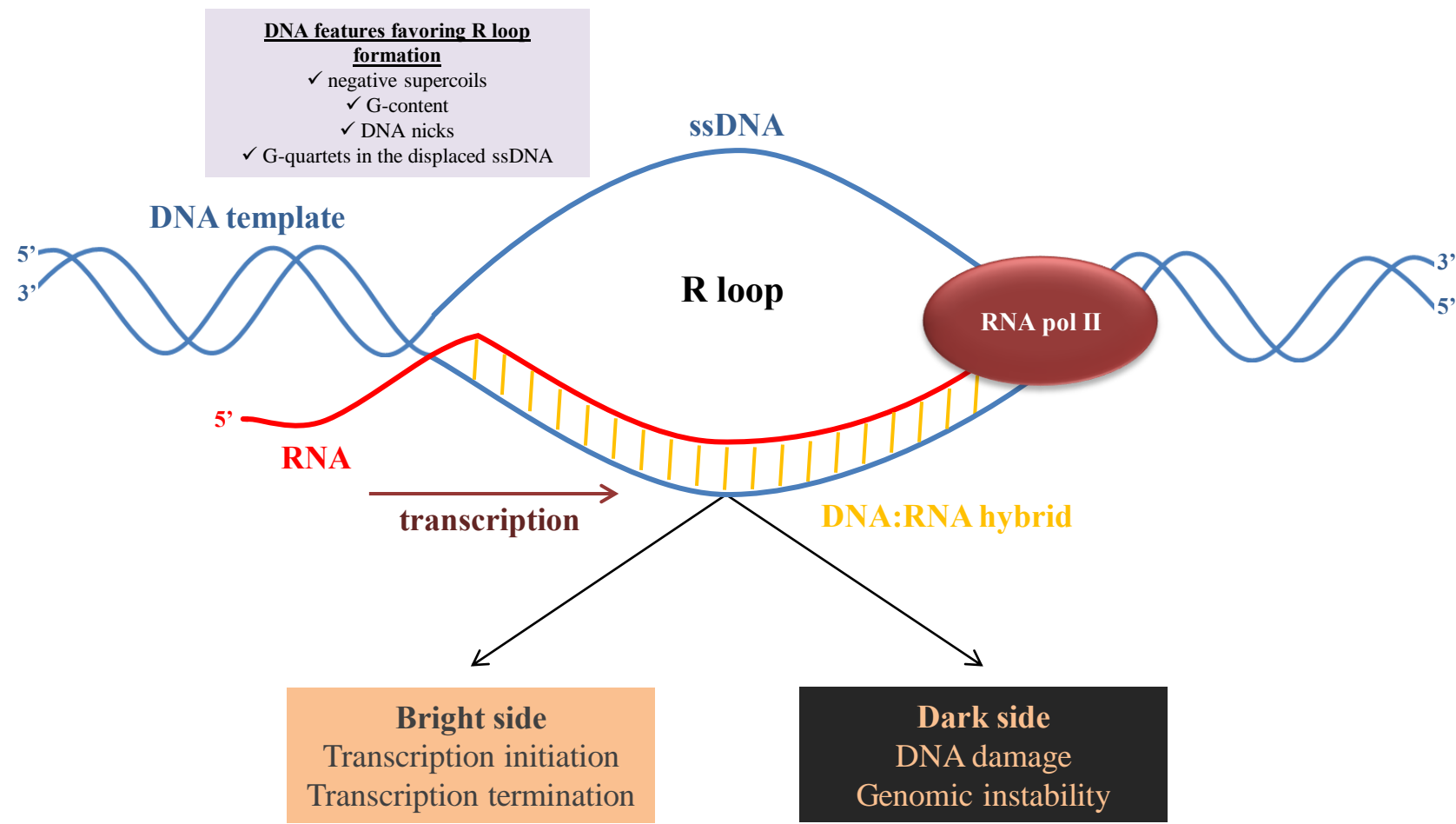

Supplement: Supplementary file 9 — Schematic presentation of an R loop. R loops are three-stranded nucleic acid structure. Factors (upper left corner) that promote R loops are indicated as well as the differential cellular effects (bottom) stemming from their formation. (PDF 557 kb) [file 12864_2017_4375_MOESM9_ESM.pdf]

Figure S7

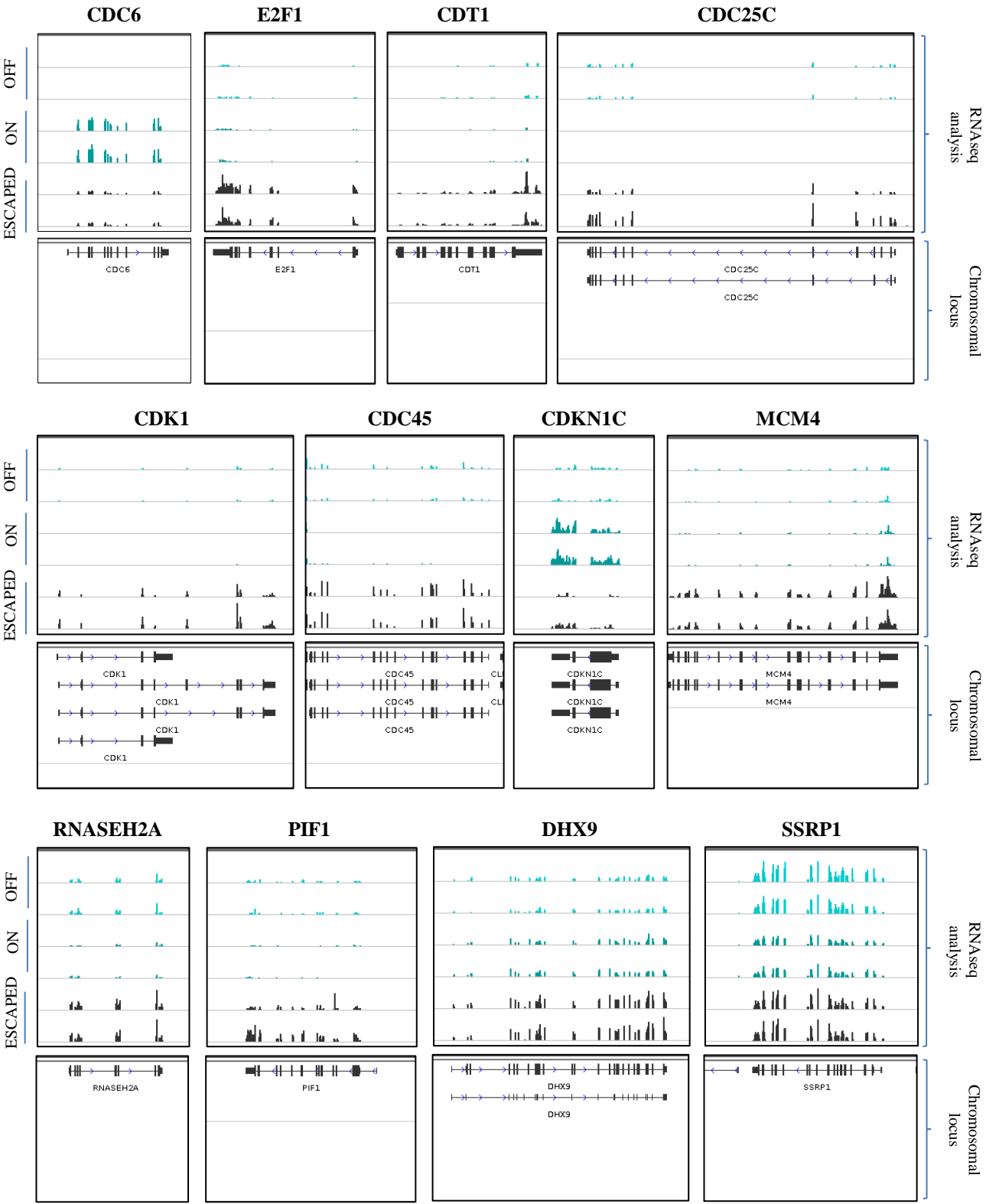

Supplement: Supplementary file 10 — Bedgraphs of indicative genes showing the specificity of RNAseq analysis. RNAseq data from two biological replicates is depicted. (PDF 47 kb) [file 12864_2017_4375_MOESM10_ESM.pdf]

Figure S8

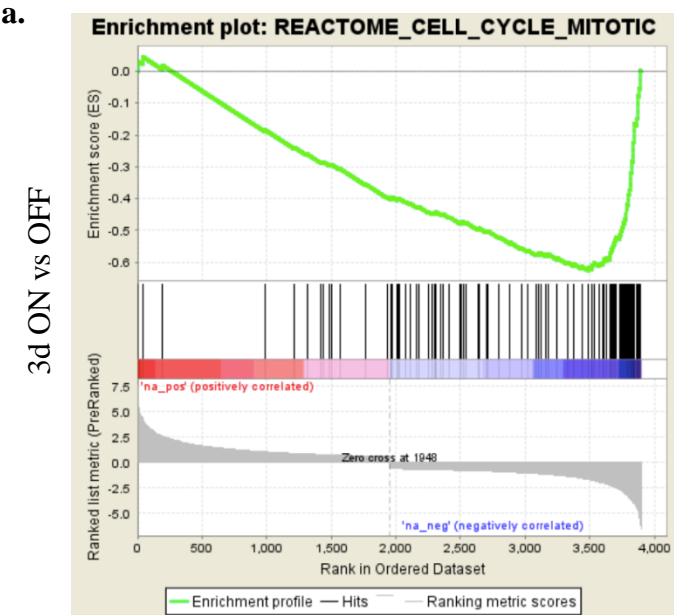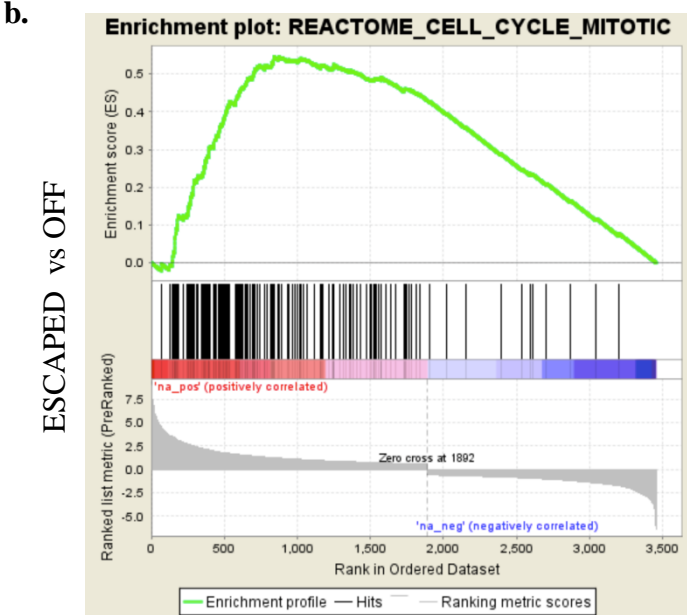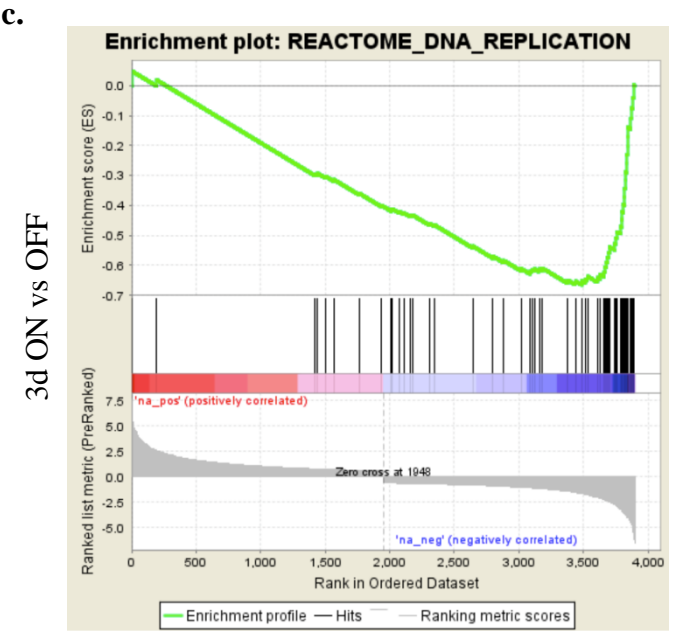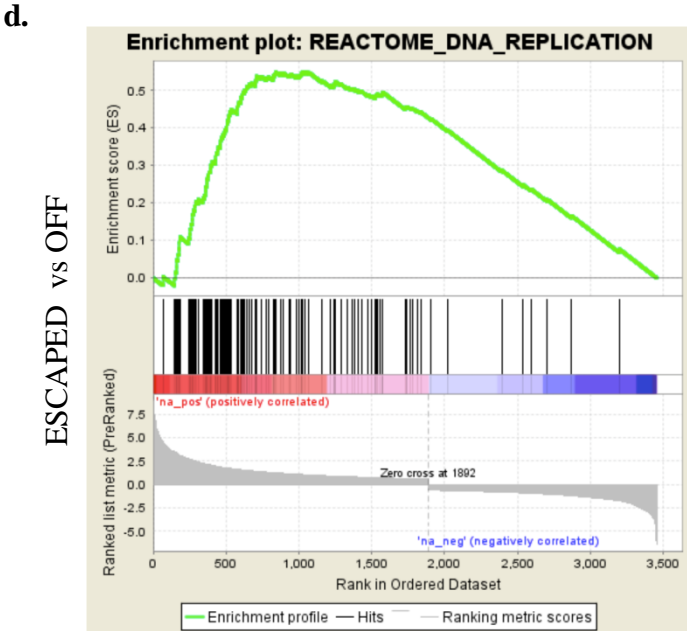

Supplement: Supplementary file 12 — Enrichment plots a-b) of the “Cell cycle mitotic” and c-d) of the “DNA replication” gene-sets. Cells entering senescence (3-day induced) showed a significant (Bonferroni-adjusted p value <0.001) down-regulation of cell-cycle and DNA replication pathways in comparison to control ones. These changes were reversed with a significant up-regulation (Bonferroni-adjusted p value <0.001) of both sets when the cells escaped from senescence. (PDF 528 kb) [file 12864_2017_4375_MOESM12_ESM.pdf]

Figure S9

HBEC CDC6 Tet-ON

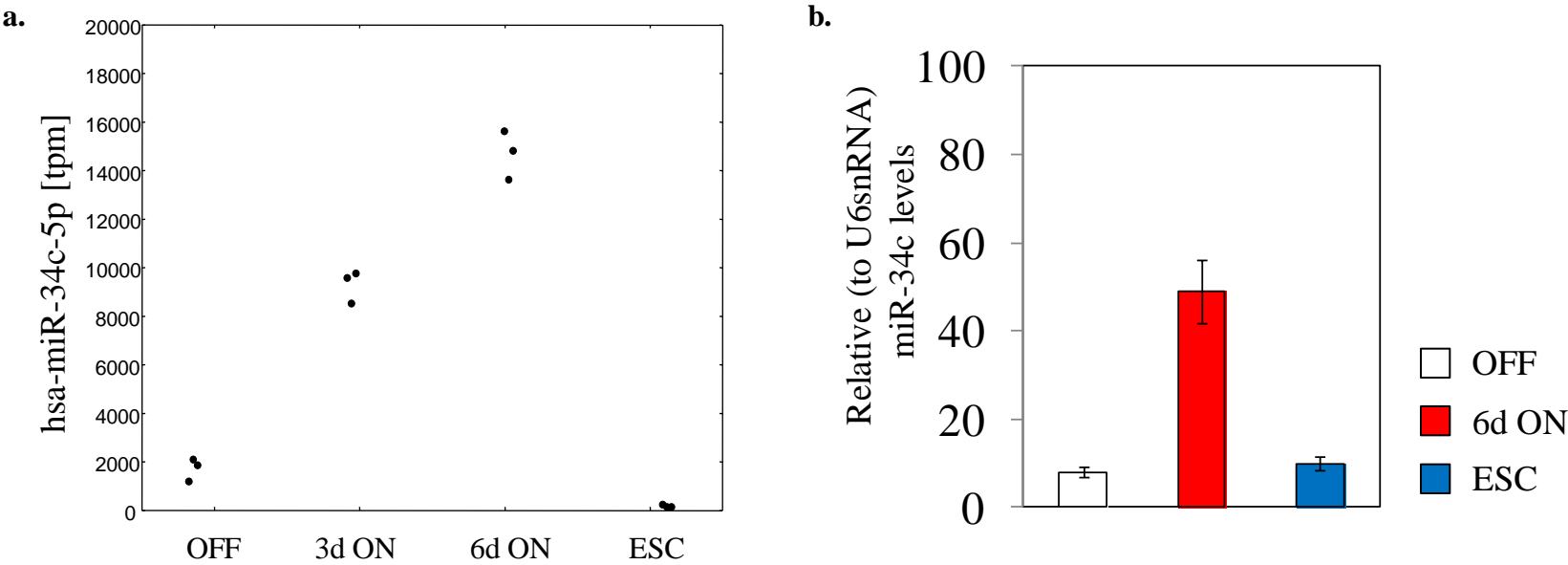

Supplement: Supplementary file 14 — miR-34c expression analysis in OFF, ON and “ESCAPED” HBEC CDC6 Tet-ON cells utilizing: a) qRT-PCR and b) miRseq analysis, tpm (transcripts per million). (PDF 25 kb) [file 12864_2017_4375_MOESM14_ESM.pdf]

Figure S10

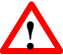  
work in a clean  
and RNAase  
free area

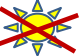  
avoid light  
exposure

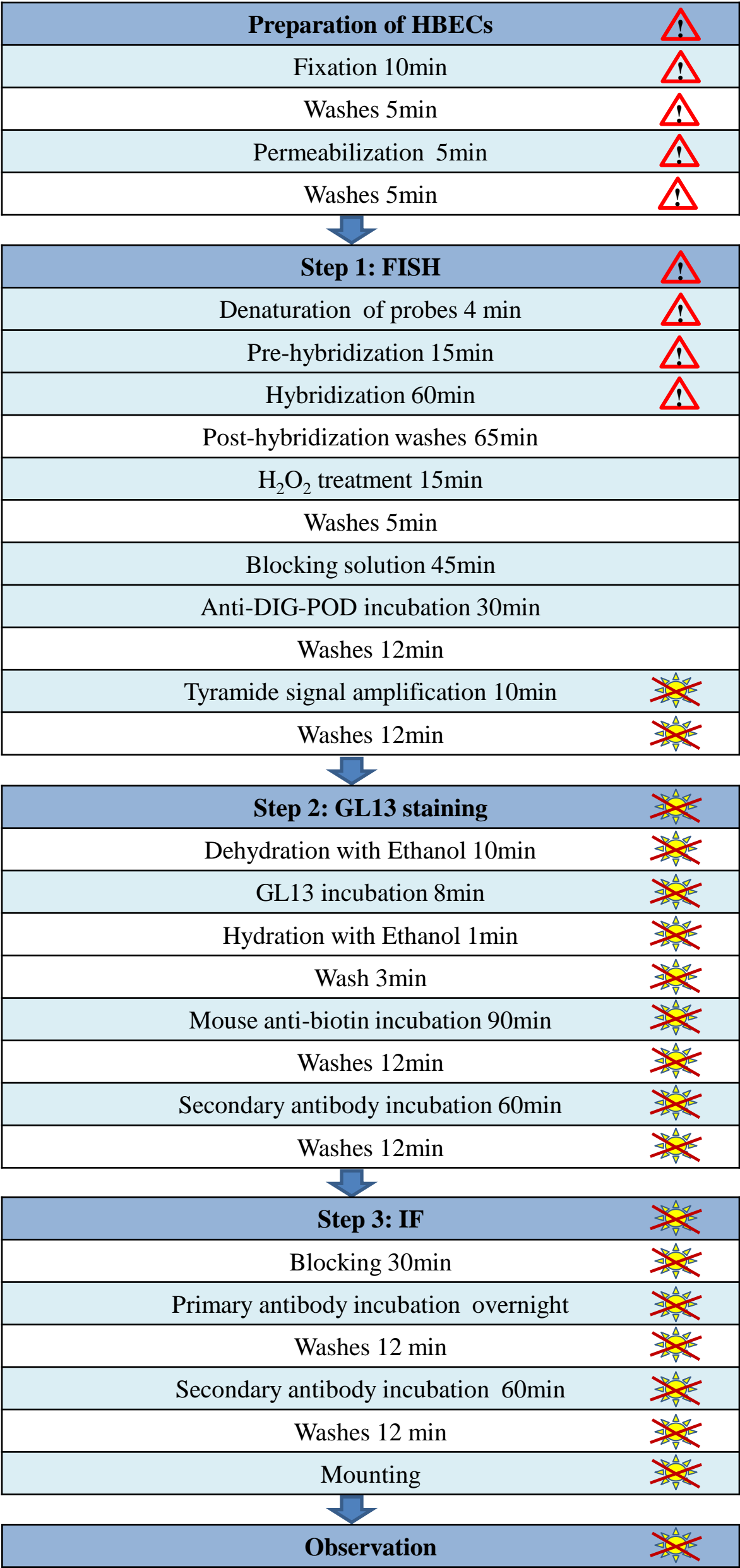

Supplement: Supplementary file 18 — Flowchart of the protocol employed to co-detect in situ gene coding (protein) and non-coding (miR) products during OIS in the HBECs CDC6 Tet-ON system. See also Additional file 17. (PDF 116 kb) [file 12864_2017_4375_MOESM18_ESM.pdf]

Figure S11

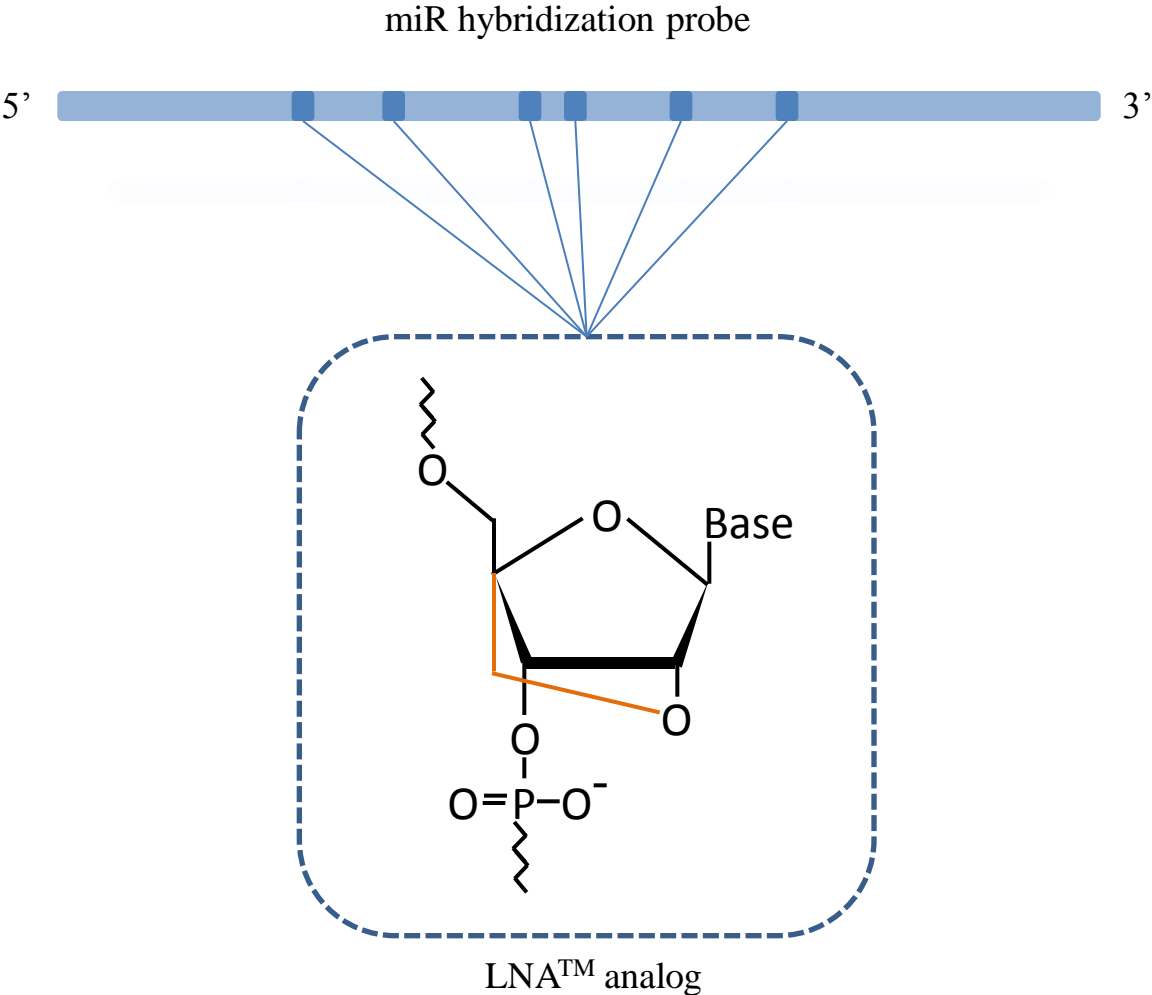

Supplement: Supplementary file 19 — Hybridization probes incorporating nucleotide analogs based on the LNA technology. (PDF 144 kb) [file 12864_2017_4375_MOESM19_ESM.pdf]

Figure S12

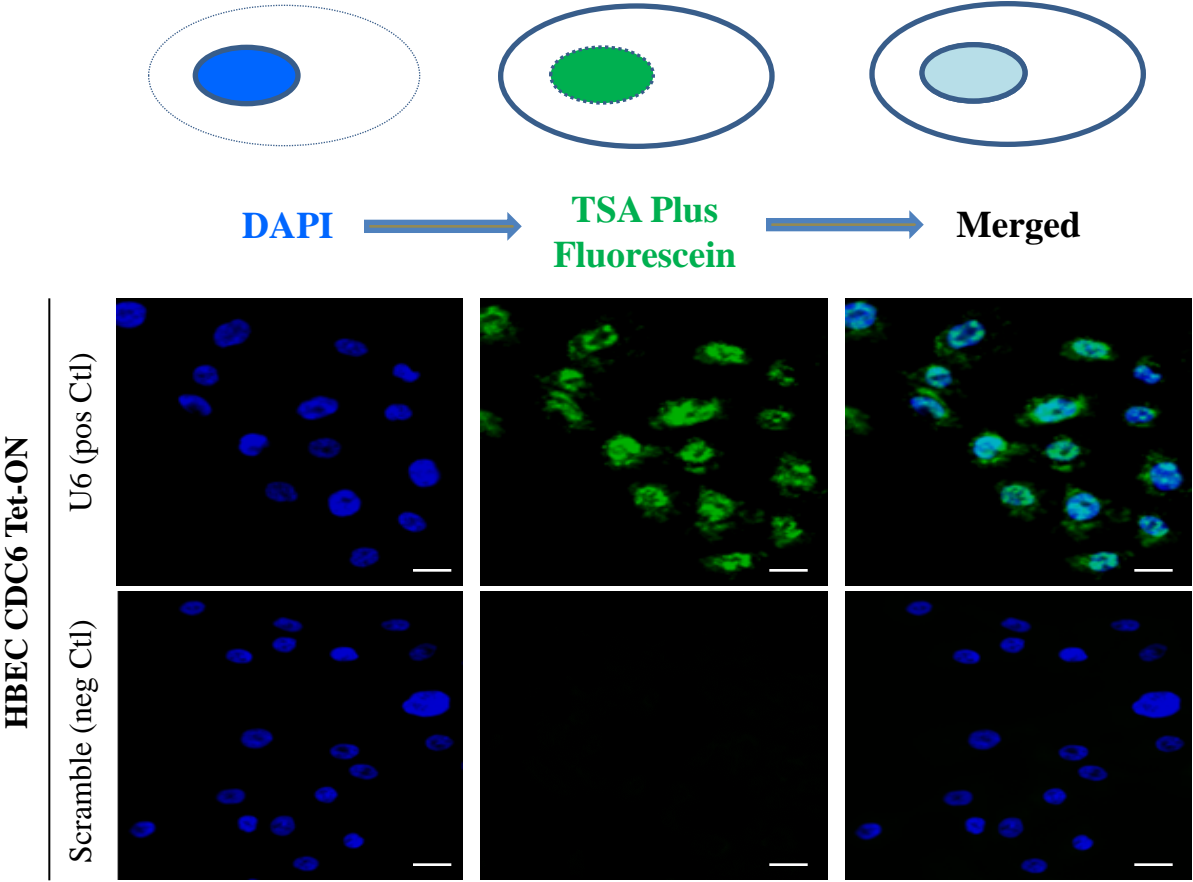

Supplement: Supplementary file 20 — Detection of U6 snRNA and scramble-miR miRNACURY control double-DIG labeled probes employing TSA plus Fluorescein system. Scale bar: 30 μm. (PDF 74 kb) [file 12864_2017_4375_MOESM20_ESM.pdf]
