# Supplementary material for: A prototypical non-malignant epithelial model to study genome dynamics and concurrently monitor micro-RNAs and proteins in situ during oncogene-induced senescence
Source: BMC Genomics. 2018 Jan 10;19:37. doi: 10.1186/s12864-017-4375-1 (PMC5763532; doi:10.1186/s12864-017-4375-1)
Supplement: Supplementary file 17 — Supplementary methods. (see also Additional file 18: Figure S10). (DOC 91 kb) [file 12864_2017_4375_MOESM17_ESM.doc]

**Appendix 1: Detailed method description (see also Suppl. Figure 10)**

**REAGENTS**

1. **Fixation of Human Bronchial Epithelial Cells (HBECs)**

Paraformaldehyde (PFA), Sigma P-6148

1. **Fluorescence In Situ Hybridization (FISH)**

Hydrogen peroxide 30% (Scharlau, Cat no: HI01351000)

Tween 20 (Sigma, Cat no P9416)

Triton X-100 (Sigma, Cat no T8787)

2x microRNA ISH Buffer (Exiqon, Cat no: 90000)

PBS tablets (Gibco by life technologies, Cat no: 18912-014)

UltraPure 20X SSC Buffer (Invitrogen, Cat no: 15557-044)

Sheep serum (Jackson Immunoresearch, Cat. No. 013-000-121)

BSA (Serva, Cat no 11930)

TSA^TM^ Plus Fluorescein System (Perkin Elmer, NEL741B001KT)

*ΜicroRNA Probes:*

hsa-miR-34c-3p (Exiqon, Cat no: 611870-360)

Scramble-miR (Exiqon, Cat no: 699004-360)

U6, hsammurno (Exiqon, Cat no: 699002-360)

*Antibodies:*

Sheep anti-DIG POD (Roche, Cat no: 11207733910)

1. **Senescence assessment**

Ethanol (EtOH) 100%, Scharlau (Cat no: ET00051000)

Compound (GL13)

*Antibodies:*

Anti-biotin antibody (Abcam, Hyb-8, ab201341)

Alexa Fluor® goat-anti-mouse (647) (ThermoFisher Scientific, Invitrogen, Cat no: A21236)

**4. Immunofluorescence (IF) analysis**

Goat serum (Invitrogen, Cat no: 31872)

SlowFade® Gold antifade reagent with DAPI (Life Technologies, Cat no: S36938)

*Antibodies:*

Polyclonal rabbit anti 53BP1 antibody (Abcam, ab21083)

Alexa Fluor® goat-anti-rabbit (568) (ThermoFisher Scientific, Invitrogen, Cat no: A11011)

**SETUP OF REAGENTS**

*4% (w/v) PFA in PBS for fixation*: Dissolve PFA powder in PBS 1x. Heat and stir the mixture until it becomes transparent. Then let the solution to cool down and adjust to pH 7.4. Store at 4 °C

*0.5% Triton X-100 in PBS 1x*: Dilute 30% Triton X-100 to 0.5% with PBS 1x. (Sterilize)

*Hydrogen peroxide (H_2_O_2_) 3% solution*: Dilute Hydrogen peroxide 30% to 3% with dH_2_O.

*PBS 1X buffer solution*: Dilute 1 PBS tablet with 500mL dH_2_O (sterilize)

*PBS-T (0.1%) buffer solution*: To 1L of PBS 1x add 1ml Tween 20.

*1X microRNA hybridization buffer*: Dilute 2X microRNA hybridization buffer with equal amount of ddH_2_O.

*5x SSC; 1x SSC; 0.2x SSC*: Dilute 20x SSC buffer to 5x, 1x and 0.2x accordingly with ddH_2_O.

*Blocking and Dilutant reagent*: preparation of both reagents is strictly based on the Instruction manual v3.0, by Exiqon (p. 13).

a) To 15 mL PBS-T (0.1%), add 300μL Sheep serum (2% final concentration). Label this tube “Blocking solution”.

b) Transfer 5ml from the tube labeled “Blocking solution” to a new tube. Label the second tube “Dilutant solution”.

c) To the tube labeled “Blocking solution”, add 300μl 30% BSA (final concentration 1%). The Blocking solution is ready to use.

d) To the tube labeled “Dilutant solution”, add 5ml PBS (final concentration 0.05% Tween and 1% sheep serum) and 330μl 30% BSA (final concentration 1%). The Dilutant solution is ready to use.

*TSA Plus working solution*: preparation is strictly based on the manual provided by PerkinElmer, NEL741001KT, p. 3.

Dilute TSA Plus stock solution in 1x Amplification Dilutent to a dilution factor 1:50. To prepare TSA Plus stock solution add 150μl HPLC grade DMSO to each vial named as “TSA Plus Amplification Reagent”.

*EtOH 70%, EtOH 50%*: dissolve EtOH 100% with dH_2_O to the appropriate final concentration.

*30% BSA, 1% BSA*: Add 30g or 1g of BSA respectively to 100ml PBS1x.

**MATERIAL AND EQUIPMENT**

Petri dishes P60 (e.g. Greiner Bio one)

Syringes Q-Max Syringe Filters (Frisenette 13mm filter, membrane 0.22μm, cat no 13CA022-100)

Sterile 1.5ml & 0.5ml micro-tubes (e.g., Sarstedt, cat. no. 72.695.500)

Sterile pipette tips (e.g Greiner bio-one, cat no: 739290)

Parafilm (Sigma-Aldrich)

Fume Hood

Micropipettes

Positively Charged Glass Slides

Hybridization oven

Coverslips

Coplin jars

PCR cycler

**PROCEDURE**

**Preparation of cells: fixation and permeabilization of HBECs**

a) Fix cells on cover slips with 4% paraformaldehyde for 10 minutes (min), at Room Temperature (RT)

b) Remove paraformaldehyde and wash two times with PBS 1x; the first one rapidly and the second one for 5 min at RT.

**Note**: Fixed cells may be kept in PBS 1x at 4^o^C for several days as long as they do not dry out.

c) Permeabilize cells by incubating with 0.5% Triton X-100 diluted in PBS 1x (stored at 4^o^C) for 5 min at 4^o^C.

**CRITICAL STEP**: Optimal penetration ensures proper binding to target (see Table I).

d) Remove 0.5% Triton X-100 and wash two times with PBS 1x; the first one rapidly and the second one for 5 min at RT.

**STEP 1: Fluorescence *In Situ* Hybridization (FISH) in HBECs**

S1.1 Pre-hybridization (optional): incubate the slides with the hybridization buffer (i.e. 1x microRNA ISH Buffer) in the absence of the probe for 15 min at the hybridization temperature (T_m_).

S1.2 Hybridization: incubate the slides with the hybridization buffer including the probe for 1 hour at the appropriate T_m_. Attention: first denature the probes by heating them at 90^0^C for 4 min, then spin down shortly and immediately add the already prepared 1x microRNA ISH buffer. Add the probe mix carefully on the pre-hybridized biological specimen. Incubation is performed in a hybridization oven under humidifying conditions. The Tm for miR34c is 56^o^C and the final concentration ranges between 50nM to 80nM (dilution factor: 1:500-1:312.5). The final concentration of U6snRNA probe is optimized by increasing concentrations ranging from 0.1nM to 1nM. The final concentration for scramble-miR is optimized according to the final concentration of the target probe. All probes are diluted in 1x microRNA ISH buffer according to Instruction manual v3.0, by Exiqon (p. 12).

**CRITICAL STEP**: Optimization of the hybridization temperature (T_m_) may start 30^o^C below the RNA Tm when detecting RNA targets. Further information regarding LNA^TM^ oligonucleotides can be found in the Instruction manual v3.0, by Exiqon (p. 23) and the corresponding website (<http://www.exiqon.com/RNA-tm>) (see Table I).

**NOTE**: After hybridization step, RNase-free conditions are no longer required.

S1.3 Post-hybridization washes: Wash biological specimens in pre-warmed SSC buffer series.

Note: After the hybridization step, RNAase free conditions are no longer required.

Caution: Take care not to dry out the samples!

a) Particularly, quickly remove excess probe by rinsing slides in pre-warmed 5x SSC buffer.

b) Wash two times with pre-warmed 5x SSC for 5 min at 4-6^o^C above the hybridization temperature (T_m_),

c) Wash two times with pre-warmed 1x SSC for 5 min at 4-6^o^ C above the hybridization temperature (T_m_),

d) Wash two times with pre-warmed 0.2x SSC for 20 min at 4-6^o^ C above the hybridization temperature (T_m_),

**CRITICAL STEP**: The stringency of post-hybridization washing depends on the Tm, the concentration of the washing buffer and the duration per wash. Proper washing decreases the stability of probe/non-specific target favoring the presence of specific hybrids (between probes-specific target) (see Table I).

S1.4 Wash two times with PBS 1x; the first one rapidly and the second one for 5 min. at RT.

S1.5 H_2_O_2_ treatment: Block endogenous peroxidase activity with 3% H_2_O_2_ for 15 min at RT (incubation is performed in the dark).

**NOTE**: Blocking of endogenous peroxidase should be performed before incubating with anti-DIG-POD (see Table I).

S1.6 Remove 3% H_2_O_2_ and wash two times with PBS 1x; the first one rapidly and the second one for 3 min at RT.

S1.7 Incubate with the blocking solution for 45 min at RT.

S1.8 Incubate with sheep anti-DIG POD at 1:4.000 in Dilutant buffer for 30 min at RT.

S1.9 Wash with PBS-T (0.1%) 1x five times; the first one rapidly and the rest ones for 3 min. each at RT.

S1.10 Incubate with TSA-plus working solution for 2 x 5 min at RT.

**NOTE**: The TSA-Plus is an up-graded version further enhancing the sensitivity than standard tyramide reagents.

S1.11 Block the reaction by washing five times in PBS-T (0.1%) 1x, the first one rapidly and the rest for 3 min at RT.

**CAUTION**: Avoid light exposure from now on!

**STEP 2: Staining with GL13 in HBECs**

S2.1 Apply EtOH 50% on the slides for 5 min at RT.

S2.2 Apply EtOH 70% on the slides for 5 min at RT.

S2.3 Apply GL13 and incubate for 8 min at 37^0^C.

**CRITICAL STEP**: Incubation with GL13 is optimized ranging from 4-10 min (see Table I).

**NOTE**: To avoid evaporation of the dye cover the biological sample (i.e. cells) during the incubation with GL13 with a slide [194].

S2.4 Rinse rapidly twice with EtOH 50% and then apply EtOH 50% for 1 min.

**CRITICAL STEP**: Longer duration of washing with EtOH 50% reduces specific staining (see Table I).

S2.5 Wash with PBS-T (0.1%) for 3 min.

S2.6 Incubate with mouse anti-biotin 1:150 in PBS 1x plus 1:40 goat serum, for 1 hour and 30 min at 37^o^C.

S2.7 Wash with PBS 1x five times; the first one rapidly and the rest one for 3 min, each at RT.

S2.8 Incubate with the secondary antibody conjugated with fluorochrome: Alexa Fluor goat anti mouse (647) at 1:200 in PBS 1x for 1 hour at RT.

S2.9 Wash with PBS 1x five times; the first one rapidly and the rest ones for 3 min, each at RT.

**STEP3: IF in HBECs**

S3.1 Block non specific epitopes: incubate with 1% BSA and 1:20 goat serum in PBS 1x for 30 min at RT

S3.2 Incubate with the primary antibody. 53BP1 antibody is diluted 1:150 (plus 1:20 goat serum) and incubated at 4^o^C overnight.

S3.3 Remove the primary antibody and wash with PBS 1x five times; the first one rapidly and the rest ones for 3 min. each at RT.

S3.4 Incubate with the secondary antibody conjugated with fluorochrome: Alexa fluor goat anti-rabbit (568), at 1:200 in PBS 1x for 1 hour at RT.

S3.5 Remove the secondary antibody wash with PBS 1x five times; the first one rapidly and the rest for 3 min, each at RT.

S3.6 Mount slides with Slow Fade® Gold antifade reagent with DAPI.

**Table I.** Troubleshooting Table.

|  | **Problem** | **Possible cause** | **Solution** | |
| --- | --- | --- | --- | --- |
| **FISH** | No or low signal | Non-sufficient penetration into cells | | Increase duration or the concentration of the buffer employed for penetration |
|  |  | Probe or target degradation | | Avoid contamination by RNases |
|  |  | Absence of target | | Choose a positive control |
|  | High background | Hybridization Temperature lower than the proper one | | Increase the Hybridization Temperature (Tm) |
|  |  | Increased concentration of probe applied | | Reduce the concentration of the probe |
|  |  | Insufficient washes | | Optimize post-hybridization washes by fine tuning the following parameters: a) temperature, b) concentration of the buffer, c) duration of washing. Up-regulation of the above parameters decreases non-specific staining at the expense of reducing specific staining. |
|  |  | Decreased incubation with blocking solution | | Increase the incubation with the blocking solution |
|  |  | Increased concentration of the anti-DIG-POD | | Decrease the concentration of the anti-DIG-POD |
|  |  | Dry out of biological specimen | | Ensure humidity during hybridization |
| **GL13 staining** | No or low signal | Non-sufficient penetration into cells | | Increase duration or the concentration of the buffer employed for penetration |
|  |  | Low duration of GL13 incubation | | Increase in a step-wise manner the incubation of GL13 |
|  |  | Reduced mouse-anti-biotin incubation | | Increase the incubation with mouse anti-biotin |
|  |  | Absence of Lipofuscin | | Choose a positive control |
|  | High background | High duration of GL13 incubation | | Reduce GL13 incubation in a step-wise manner |
|  |  | Decreased washes | | Increase EtOH and PBS1x washes after GL13 incubation |
| **IF** | No or low signal | Non-sufficient penetration into cells | Increase duration or the concentration of the buffer employed for penetration | |
|  |  | Low concentration of primary antibody | | Increase concentration of primary antibody |
|  |  | Absence of target | | Choose a positive control |
|  | High background | Insufficient blocking | | Increase the duration of blocking solution, or change the blocking medium |
|  |  | Increased concentration of secondary antibody | | Reduce the concentration of the secondary antibody |
|  |  | Decreased washes | | Increase the washes |
